# A Fish Leukocyte Immune-Type Receptor Uses a Novel Intracytoplasmic Tail Networking Mechanism to Cross-Inhibit the Phagocytic Response

**DOI:** 10.3390/ijms21145146

**Published:** 2020-07-21

**Authors:** Chenjie Fei, Myron A. Zwozdesky, James L. Stafford

**Affiliations:** Department of Biological Sciences, University of Alberta, Edmonton, AB T6G 2E9, Canada; chenjie@ualberta.ca (C.F.); myronz@ualberta.ca (M.A.Z.)

**Keywords:** comparative immunology, phagocytosis, cross-talk, intracellular signaling, immunoregulatory receptors, innate immunity, vertebrates, fish

## Abstract

Channel catfish (*Ictalurus punctatus*) leukocyte immune-type receptors (IpLITRs) are a family of immunoregulatory proteins shown to regulate several innate immune cell effector responses, including phagocytosis. The precise mechanisms of IpLITR-mediated regulation of the phagocytic process are not entirely understood, but we have previously shown that different IpLITR-types use classical as well as novel pathways for controlling immune cell-mediated target engulfment. To date, all functional assessments of IpLITR-mediated regulatory actions have focused on the independent characterization of select IpLITR-types in transfected cells. As members of the immunoglobulin superfamily, many IpLITRs share similar extracellular Ig-like domains, thus it is possible that various IpLITR actions are influenced by cross-talk mechanisms between different IpLITR-types; analogous to the paired innate receptor paradigm in mammals. Here, we describe in detail the co-expression of different IpLITR-types in the human embryonic AD293 cell line and examination of their receptor cross-talk mechanisms during the regulation of the phagocytic response using imaging flow cytometry, confocal microscopy, and immunoprecipitation protocols. Overall, our data provides interesting new insights into the integrated control of phagocytosis via the antagonistic networking of independent IpLITR-types that requires the selective recruitment of inhibitory signaling molecules for the initiation and sustained cross-inhibition of phagocytosis.

## 1. Introduction

Classically performed by macrophages, phagocytosis is an evolutionarily conserved innate protective mechanism that results in the destruction of microbial intruders, and it is also a cellular process that facilitates the removal of dead cells from the host following infection/inflammation-mediated tissue damage [1]. Macrophages can recognize a dynamic array of microbial signatures as well as microbes that are coated with opsonins via engagements of various surface-expressed immunoregulatory receptor-types [2]. The integration of multiple sub-membrane proximal signaling events from different receptor-types, generally termed cross-talk, provides input information to immune cells about the chemical and physical characteristics of extracellular targets, which ultimately controls subsequent cellular responses [3,4,5,6]. For instance, co-engagement of toll-like receptors (TLRs) with Fc receptors (FcRs) synergistically augments the secretion of pro-inflammatory cytokines, which are only minimally produced when either of these receptors are activated independently [7,8]. However, excessive production of pro-inflammatory cytokines and highly reactive tissue-damaging products (e.g., reactive oxygen species) during phagocytosis is detrimental to host tissues, thus it is no surprise that this process is strictly regulated to mitigate unnecessary collateral host damage [9].One key regulatory mechanism controlling innate immune cell effector responses during inflammation is antagonistic receptor cross-talk. For example, activating receptors (e.g., FcγRIIA) are often co-crosslinked with inhibitory counterparts (e.g., FcγRIIB) during the phagocytic process [10]. This results in the recruitment of inhibitory signaling molecules (e.g., SHIP, SHP-1/2 phosphatases) to sites of cell-target contact (i.e., the phagocytic synapse) that serve to down-regulate phagocytosis and control the secretion of bioactive and antimicrobial molecules [10,11,12]. The loss of inhibitory FcγRs in knock-out animal models is associated with autoimmune diseases as a consequence of the dysregulated secretion of pro-inflammatory molecules (e.g., cytokines), thus reinforcing the importance of antagonistic receptor cross-talk during the control of inflammatory responses [13,14]. Overall, the simultaneous activation of multiple immunoregulatory receptor-types and the integration of their various signaling abilities is an important regulatory mechanism required for modulating the overall magnitude of innate immune cell-mediated antimicrobial activities [4]. However, the mechanistic details responsible for regulating antimicrobial responses, such as phagocytosis, are not fully understood and studies using alternative vertebrate models may provide new insights into conserved and divergent aspects of receptor cross-talk mechanisms.

To better understand conserved and divergent aspects of receptor-mediated signaling events during the control of innate immune cell effector responses, our research has focused on the functional characterization of channel catfish (*Ictalurus punctatus*) leukocyte immune-type receptors (IpLITRs). These diverse immunoregulatory receptor proteins share basic structural and distant phylogenetic relationships with several important mammalian immunoregulatory receptors including FcRs, FcR-like proteins (FcRLs), leukocyte Ig-like receptors (LILRs), and killer cell Ig-like receptors (KIRs) [15,16], as well as an ability to network phosphotyrosine-dependent intracellular signaling [17,18,19,20]. Although endogenous ligands for IpLITRs are unknown, heterologous over-expression of epitope-tagged recombinant IpLITRs has proven to be a valuable model for investigating the dynamic signaling potentials of these diverse teleost proteins [17,18,19,20,21,22,23,24,25,26]. For example, IpLITR 2.6b (termed 2.6b^ITAM CYT^ throughout this study) associates with immunoreceptor tyrosine-based activation motif cytoplasmic tail (ITAM CYT)-containing adaptor molecules and activates phagocytosis by engaging a similar profile of signaling mediators (e.g., Syk, Erk1/2) that also regulate the classical mammalian phagocytic pathway [22]. Conversely, IpLITR 1.1b (termed as 1.1b^WT CYT^) contains a long CYT region with several tyrosine residues and two immunoreceptor tyrosine-based inhibitory motifs (ITIMs). This receptor also displays multifunctional signaling capabilities including both inhibitory and stimulatory actions on innate immune cell responses [18,19]. Since both stimulatory and inhibitory IpLITR-types are co-expressed by catfish immune cells, integrated signaling between these proteins is likely important for their overall immunoregulatory functions. Putative stimulatory and inhibitory IpLITR-types also share membrane distal Ig-like domains with high amino acid sequence similarities, which suggests that they may engage common ligands that could augment their co-engagement and immunoregulatory cross-talk potential [15,16].

In the present study, we show that the co-expression of differentially epitope-tagged IpLITR constructs in the human epithelioid AD293 cell line is a valuable tool for functionally examining and biochemically characterizing IpLITR cross-talk signaling events. Overall, our data support a new regulatory capability of IpLITR 1.1b as a potent inhibitor of the IpLITR 2.6b/IpFcRγ-L-mediated phagocytic response. Specifically, we show that 1.1b^WT CYT^-mediated cross-inhibitory activity occurs by preventing the recruitment of and/or diminishing the phosphorylation status of intracellular signaling molecules during initial stages of target engulfment (i.e., within phagocytic cups). Co-crosslinking with 2.6b^ITAM CYT^ is a prerequisite for the ability of 1.1b^WT CYT^ to produce inhibitory signals; supporting that stimulatory signaling activity is required to initiate subsequent inhibitory pathways. Finally, we reveal that distinct regions of the IpLITR 1.1b CYT are required for the coordinated recruitment of C-terminal Src kinase (Csk) and Src homology 2-containing protein tyrosine phosphatase 2 (SHP-2) that work in concert to sustain the inhibition of phagocytosis and reduce activation of the extracellular signal-regulated kinase 1/2 (Erk1/2) signaling pathway. Taken together, this represents a novel mechanism for the cross-inhibition of phagocytosis and suggests that immunoregulatory receptor cross-talk is likely an important regulatory mechanism utilized by IpLITRs to fine-tune innate immune cell effector responses in fish.

## 2. Results

### 2.1. Examination of Independent Construct Activity in IpLITR Co-Expressing (2.6b^ITAM CYT^/1.1b^WT CYT^) AD293 Cells Using Epitope Tag-Specific mAb-Opsonized Beads

**Figure 1:**
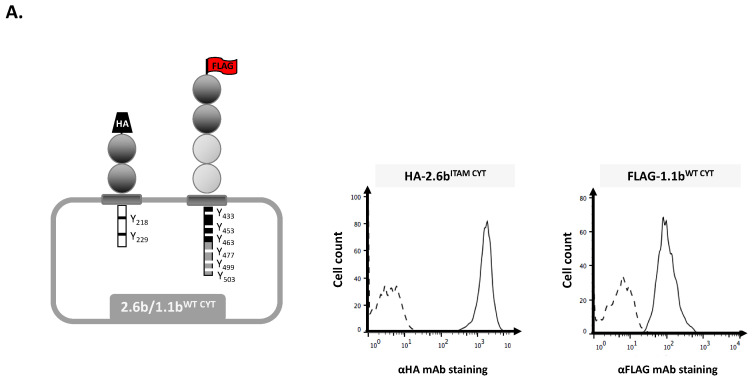

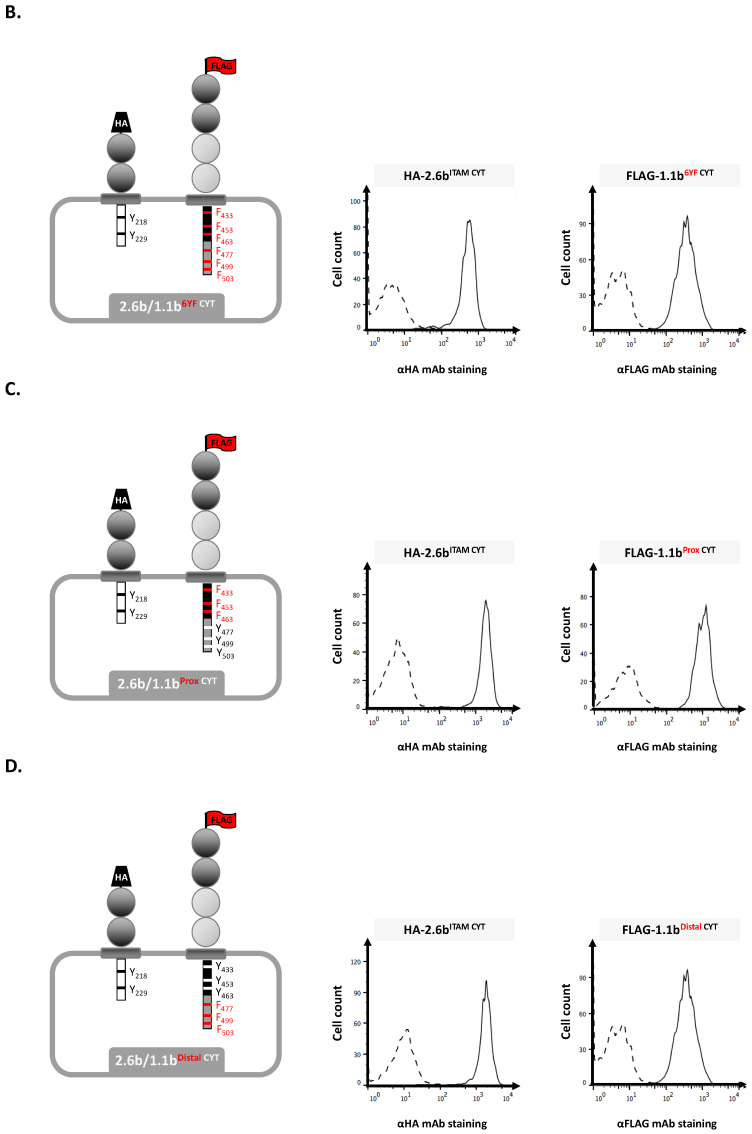

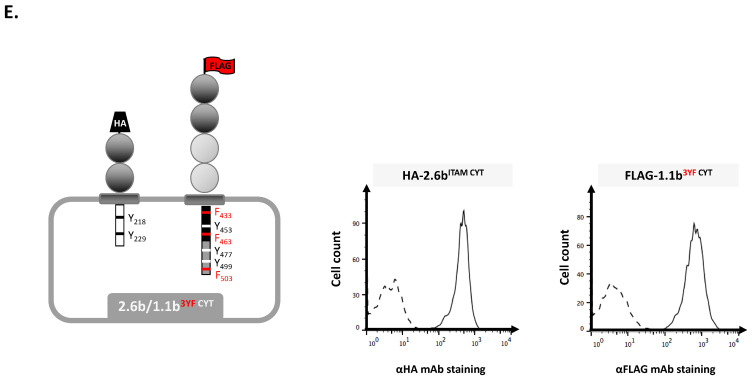
Leukocyte immune-type receptors (IpLITR) co-expressing AD293 cell lines used in this study. Schematic representation of the various N-terminal epitope-tagged IpLITR constructs and their surface expression profiles are displayed for 2.6b^ITAM CYT^/1.1b^WT CYT^ (**A**), 2.6b^ITAM CYT^/1.1b^6YF CYT^ (**B**), 2.6b^ITAM CYT^/1.1b^Prox CYT^ (**C**), 2.6b^ITAM CYT^/1.1b^Distal CYT^ (**D**), and 2.6b^ITAM CYT^/1.1b^3YF CYT^ (**E**). Note: red letters in the schematics indicate a mutant construct with the specific positions of each tyrosine (Y) to phenylanaline (F) mutations shown in red when compared to the non-mutated 1.1b WT construct shown in (A).

To investigate IpLITR-mediated cross-talk regulation of the phagocytic process, we generated a series of AD293 cell lines co-expressing N-terminal hemagglutinin (HA) and FLAG epitope-tagged receptor constructs (Figure 1). This allowed receptor-specific (i.e., HA-2.6b^ITAM CYT^ and FLAG-1.1b^WT CYT^) co-crosslinking using microbead targets co-opsonized with anti-HA and -FLAG epitope tag-specific monoclonal antibodies (mAb), respectively. To verify that each IpLITR construct could be independently engaged in this co-expression system, yellow-green (YG) fluorescent beads opsonized with various concentrations of α-HA mAb (i.e., 0.16–1.28 µg/mL, Cedarlane Laboratories Ltd.; Ontario, Canada) or α-FLAG mAb (i.e., 0.32–5 µg/mL) were used to trigger the constructs on 2.6b^ITAM CYT^/1.1b^WT CYT^ co-expressing AD293 cells. When engaged with the α-HA mAb, only opsonized beads (to trigger 2.6b^ITAM CYT^), 2.6b^ITAM CYT^/1.1b^WT CYT^ AD293 cells significantly activated their phagocytic activity after 15 min (Figure S1; left panel). Specifically, 12.6% of the cells displayed phagocytic activity (i.e., % cells with at least one completely engulfed target) after 15 min when they were incubated with 0.16 µg/mL of α-HA mAb-opsonized YG beads. Higher concentrations of the α-HA opsonizing antibody increased the phagocytic activity of the cells to 33.3% (0.32 µg/mL), 44.7% (0.64 µg/mL), and 41.9% (1.28 µg/mL). Comparatively, at 30 min, the phagocytic activity of the cells increased to 34.5%, 55.4%, 61.4%, and 58% when using α-HA mAb-opsonized beads (0.16–1.28 µg/mL), respectively (Figure S1; left panel). At 15 min, the bead-binding activity of the cells ranged between 10.9% and 16.4% (indicated in grey on the bar graphs) and at 30 min the binding activity was <10% when 2.6b^ITAM CYT^/1.1b^WT CYT^ co-expressing AD293 cells were incubated with α-HA mAb only opsonized beads (Figure S1; left panel, grey are on the bar graphs). No phagocytic activity was observed when the cells were incubated with isotype IgG1 beads (Figure S1).

In comparison, when the 2.6b^ITAM CYT^/1.1b^WT CYT^ co-expressing AD293 cells were incubated with α-FLAG mAb only opsonized beads (to trigger 1.1bWT CYT), they had negligible phagocytic activity and only at the highest concentrations of the α-FLAG mAb tested (i.e., 2.5 µg/mL and 5.0 µg/mL) did the cells associate with opsonized beads at a comparable level to what we observed using 0.64 µg/mL and 1.28 µg/mL of α-HA mAb only opsonized beads (i.e., ~50% gated; Figure S1). Specifically, at 15 min, 2.5 µg/mL of α-FLAG mAb only opsonized YG beads induced only 4.6% phagocytic activity but these cells had 43.4% bead-binding activity. Similarly, at 5 µg/mL, 6.3% phagocytic activity and 41.6% bead-binding activity was observed at 15 min (Figure S1; left panel). This trend continued at 30 min but with slight increases in phagocytic and binding activities observed at the highest concentrations of α-FLAG mAb only opsonized YG beads tested (Figure S1; right panel). These data demonstrate that different phagocytic phenotypes can be observed by the independent crosslinking of IpLITR constructs on IpLITR co-expressing AD293 cells. As expected, 2.6b^ITAM CYT^ triggers a predominant phagocytic phenotype, whereas 1.1b^WT CYT^ preferentially binds to but does not readily phagocytose opsonized targets. 

Prior to performing the co-crosslinking experiments, titration of the respective mAbs used for bead co-opsonization was performed and included an additional cell line co-expressing 2.6b^ITAM CYT^ and a tyrosine to phenylalanine (YF) mutant (i.e., the functionally deficient 1.1b^6YF CYT^) construct to control for any possible non-phosphotyrosine-dependent inhibitory signals. Specifically, 2.6b^ITAM CYT^/1.1b^WT CYT^ and 2.6b^ITAM CYT^/1.1b^6YF CYT^ co-expressing AD293 cells were incubated with α-HA and α-FLAG mAb opsonized YG beads for 60 min at 4 °C to inhibit phagocytosis but still allow for bead-binding activity (Figure S2). After 60 min, ~22.8% binding activity was observed when 2.6b^ITAM CYT^/1.1b^WT CYT^ cells were incubated with 0.16 µg/mL of α-HA mAb only opsonized YG beads; this value gradually increased to 37.4%, 44.3%, and 49.9% with increasing concentrations of the α-HA mAb (Figure S2A). The crosslinking of 1.1b^WT CYT^ using different concentrations of α-FLAG mAb only opsonized YG beads also showed increased binding activity at increasing concentrations of α-FLAG mAb (Figure S2A). When 2.6b^ITAM CYT^/1.1b^6YF CYT^ cells were incubated with various opsonized YG beads, similar trends were observed in their binding profile (Figure S2B). For example, cells incubated with α-HA mAb only opsonized beads at increasing (i.e., 0.16–1.28 µg/mL) concentrations had 14.2%, 35.1%, 40.8%, and 44.4% binding activities. Similarly, the crosslinking of 1.1b^6YF CYT^ using increasing concentrations of α-FLAG mAb opsonized YG beads also showed a correlative increase in their target binding activity (Figure S2B). Overall, these binding profiles allowed us to determine the optimal concentrations of α-HA and α-FLAG mAbs required to co-opsonize YG beads for co-crosslinking studies. Accordingly, we selected 0.32 µg/mL of α-HA and 2.5 µg/mL of α-FLAG concentrations for co-engaging IpLITR constructs. To further show that these mAb concentrations achieved similar binding profiles, YG beads were co-opsonized with 0.32 µg/mL of α-HA mAb and 2.5 µg/mL of isotype IgG1 or 2.5 µg/mL of α-FLAG mAb and 0.32 µg/mL of isotype IgG1. The addition of isotype IgG1 ensures that the same overall concentrations of IgG proteins are present on the different beads. After a 60 min incubation at 4 °C, the binding activities of independent and co-crosslinked constructs were examined (Figure S2C). Overall, no significant differences in bead-binding activities were observed regardless of the mAb-opsonized beads used.

### 2.2. Co-Crosslinking 2.6b^ITAM CYT^ with 1.1b^WT CYT^ Inhibits the ITAM-Mediated Phagocytic Response in IpLITR Co-Expressing AD293 Cells

**Figure 2:**
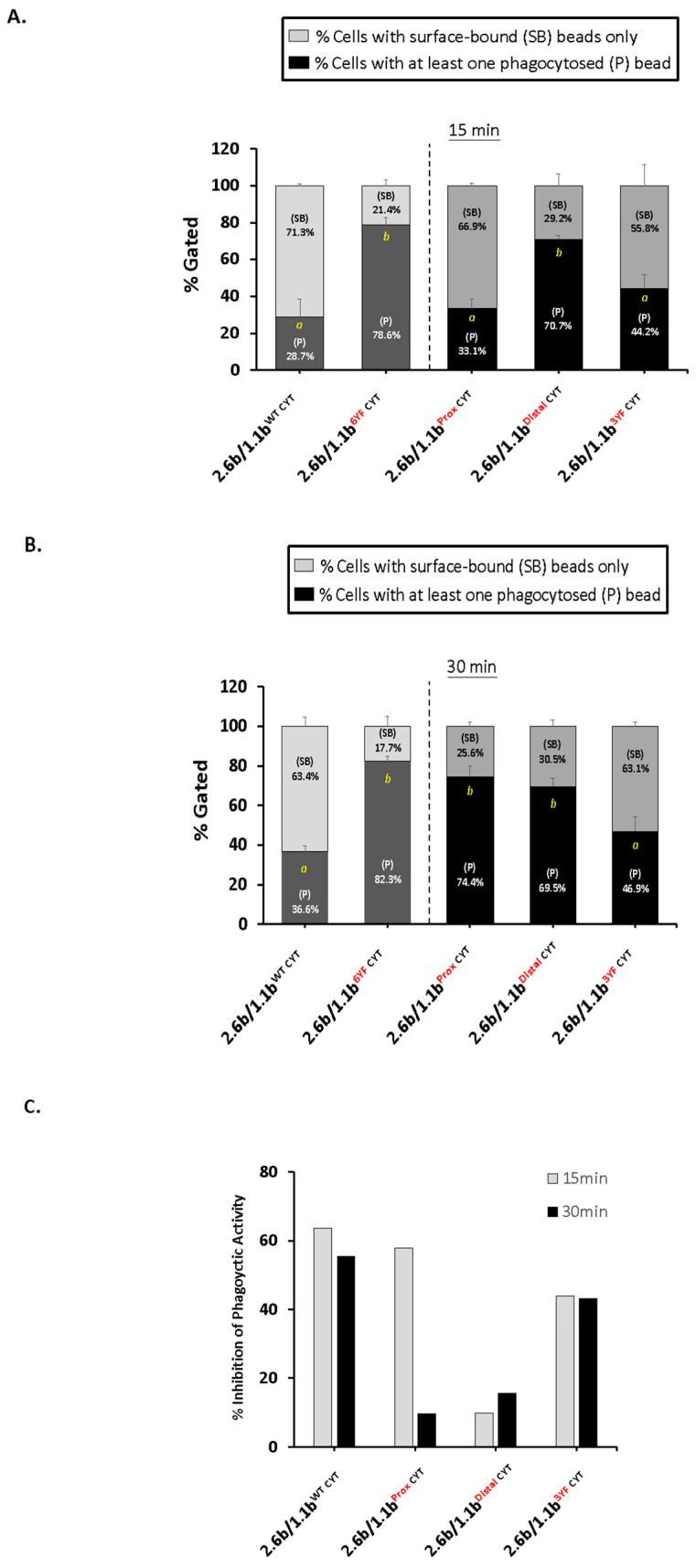
Csk-binding motif (tyrosine 453) and immunoreceptor tyrosine-based inhibitory motifs (ITIMs, tyrosine 477 and 499) in the cytoplasmic region of 1.1b^WT CYT^ are required to sustain the inhibition of 2.6b^ITAM CYT^-mediated phagocytosis. AD293 cells (2 × 10^5^) co-expressing 2.6b^ITAM CYT^ and various 1.1b^CYT^ constructs (i.e., 1.1b^WT CYT^, 1.1b^6YF CYT^, 1.1b^Prox CYT^, 1.1b^Distal CYT,^ and 1.1b^3YF CYT^) were incubated with 4.5 µm yellow-green beads (6 × 10^5^) opsonized with α-HA and α-FLAG mAbs for 15 min (**A**) and 30 min (**B**) at 37 °C prior to the analysis of samples using the ImageStream X Mark II instrument. Cells with only surface-bound (SB) targets (light grey bars) or with ≥1 phagocytosed (P) bead (black bars) were identified and gated as reported [27]. The percentage of cells displaying the two phenotypes were calculated as: % cells with (P) bead or % cells with (SB) beads only / % total cells associated with beads. Each bar represents the mean ± SEM of at least three independent experiments. Differing letters (‘a’ or ‘b’) indicate statistical significance (*p* ≤ 0.05) between % phagocytosis means. Experimental groups were compared using a one-way ANOVA, followed by the Tukey test using Prism 6 software (GraphPad Software, La Jolla, CA, USA). (**C**) Percent inhibition of phagocytosis was normalized relative to the value of the 2.6b^ITAM CYT^/1.1b^6YF CYT^ control group and was calculated as: [% phagocytosis of 2.6b^ITAM CYT^/1.1b^6YF CYT^ (maximal % phagocytosis) – (% phagocytosis of each group) / (% phagocytosis of 2.6b^ITAM CYT^/1.1b^6YF CYT^]. Percent inhibition of phagocytosis at 15 min and 30 min was then graphed as white and black bars, respectively. Note: red letters in y-axis labels of each graph indicates the names of each mutant construct tested.

To investigate IpLITR-mediated cross-talk regulation of the phagocytic response, we co-crosslinked 2.6b^ITAM CYT^ with various 1.1b^CYT^ constructs using 4.5 µm YG beads co-opsonized with 0.32 µg/mL of α-HA mAb and 2.5 µg/mL of α-FLAG mAb and then examined their phagocytic activities using an ImageStream X flow cytometer (Figure 2). At 15 min, co-crosslinking of 2.6b^ITAM CYT^ with 1.1b^WT CYT^ significantly inhibited the phagocytic response (Figure 2A). Specifically, as shown on the left side of the dashed line, 78.6% of 2.6b^ITAM CYT^/1.1b^6YF CYT^ co-expressing cells were phagocytic compared to the 28.7% phagocytic activity observed when 2.6b^ITAM CYT^/1.1b^WT CYT^ co-expressing cells were co-crosslinked (Figure 2A). This clearly shows that 2.6b^ITAM CYT^-mediated phagocytosis is significantly inhibited (i.e., ~64% reduction in phagocytic activity) when it is co-crosslinked with the intact tyrosine-containing CYT region of IpLITR 1.1b. At 30 min, a sustained inhibition of the 2.6b^ITAM CYT^-mediated phagocytic response by co-crosslinking with 1.1b^WT CYT^ was observed (i.e., ~55% inhibition of phagocytosis; Figure 2B). 

To identify the CYT region(s) required for IpLITR 1.1b-mediated cross-inhibition of phagocytosis, three additional constructs were tested: 1.1b^Prox CYT^, 1.1b^Distal CYT^, and 1.1b^3YF CYT^ (see Figure 1C–E for detailed information regarding these constructs). When co-crosslinked with 2.6b^ITAM CYT^, the 1.1b^Prox CYT^ construct containing mutated tyrosines at residue positions 433, 453, and 463 (all located within the membrane proximal region), significantly inhibited (~58% inhibition) phagocytosis at 15 min (Figure 2A). However, this effect was not sustained at 30 min since the phagocytic activity of the 2.6b^ITAM CYT^/1.1b^Prox CYT^ co-expressing cells rebounded back to 74.4%, which was not significantly different from the 2.6b^ITAM CYT^/1.1b^6YF CYT^ co-expressing cell phagocytic activity (Figure 2B). This suggests that an intact ITIM-containing distal CYT region of IpLITR 1.1b is required for the initiation but not sustainment of the inhibitory response. Conversely, when co-crosslinked with 2.6b^ITAM CYT^, the 1.1b^Distal CYT^ construct containing mutated tyrosines at positions 477, 499 (each within ITIMs), and 503 (within an immunoreceptor tyrosine-based switch motif; ITSM), failed to inhibit 2.6b^ITAM CYT^-mediated phagocytosis at 15 min and 30 min (Figure 2), thus verifying the requirement of an intact distal ITIM-containing CYT region for inhibitory activity. When the 1.1b^3YF CYT^ construct containing intact tyrosines at positions 453, 477, and 499 was tested for cross-inhibiting activity, like 1.1b^WT CYT^, it significantly inhibited phagocytic activity at 15 min (43.8% inhibition), which was also sustained after 30 min (43.1% inhibition; Figure 2). This shows that tyrosines 453, 477, and 499 are indispensable for the initiation and sustainment of IpLITR 1.1b-mediated cross-inhibition of ITAM CYT-triggered phagocytosis, whereas tyrosines 477 and 499 are only functionally capable of initiating the inhibitory response. Figure 2C summarizes the % inhibitory activities of the various constructs at 15 min and 30 min when normalized to the activity observed using the 2.6b^ITAM CYT^/1.1b^6YF CYT^ co-expressing AD293 control cells. Of note is the failure of the 1.1b^Prox CYT^ construct to sustain its inhibitory effect at 30 min.

### 2.3. Co-Crosslinking 2.6b^ITAM CYT^ with 1.1b^WT CYT^ Significantly Abrogates 2.6b^ITAM CYT^-Induced Intracellular Phosphotyrosine Levels

To examine the effect of 1.1b^WT CYT^-mediated cross-talk inhibition on the 2.6b^ITAM CYT^-mediated activation of intracellular phosphotyrosine signaling events, an imaging flow cytometry-based phospho-flow assay was developed. Specifically, 2.6b^ITAM CYT^/1.1b^WT CYT^ co-expressing cells were incubated with light-yellow (LY) beads co-opsonized with 0.32 µg/mL of α-HA mAb and 2.5 µg/mL of isotype IgG1. After 15 min at 37°C, cells were immediately fixed, permeabilized, and intracellularly stained using an Alexa 488-conjugated α-phosphotyrosine mAb. Two cell populations (i.e., cells associating or not with the LY beads) were then gated based on their LY fluorescent signals and then analyzed for phosphotyrosine staining intensities (Figure S3A). To measure the intensity of phosphotyrosine signals within individual cells, an intensity mask was used to identified signals with mean fluorescent intensity (MFI) values above 72 to set the threshold above background signals (Figure S3B). After applying this mask to the cell populations of interest, cells without target bead associations showed basal levels of phosphotyrosine signals (MFI = 143) and, predictably, an enhanced signal intensity (MFI = 2781) was observed when the IpLITR-expressing cells bound α-HA mAb opsonized LY beads (Figure S3C). Since this phospho-flow assay detects increased phosphotyrosine signals following 2.6b^ITAM CYT^ activation, we used this as a platform to further investigate the impact of IpLITR-mediated cross-talk inhibition on intracellular phosphotyrosine levels.

**Figure 3:**
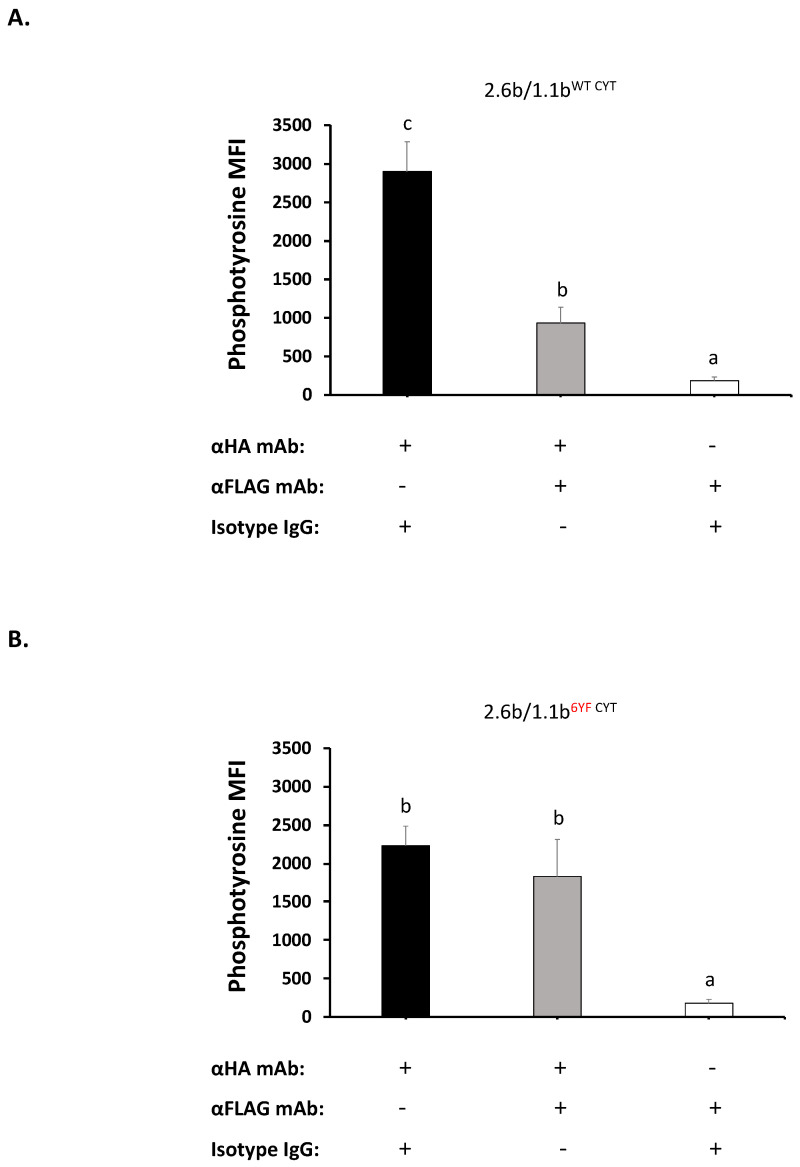
Down-regulation of 2.6b^ITAM CYT^-mediated phosphotyrosine signaling following co-crosslinking with 1.1b^WT CYT^. AD293 cells (2 × 10^5^) co-expressing 2.6b^ITAM CYT^/1.1b^WT CYT^ (**A**) and 2.6b^ITAM CYT^/1.1b^6YF CYT^ (**B**) were incubated with 3.55 µm light-yellow (LY) beads (6 × 10^5^). LY beads were opsonized with α-HA and α-FLAG mAbs to co-crosslink 2.6b^ITAM CYT^ and 1.1b^CYT^ constructs, respectively. To independently crosslink 2.6b^ITAM CYT^ or 1.1b^CYT^ constructs, LY beads were opsonized with α-HA or α-FLAG mAbs alone, respectively. After 15 min at 37 °C, cells were permeabilized and intracellularly stained with an Alexa 488-conjugated α-phosphotyrosine mAb and cells were then analyzed using an ImageStream X Mark II instrument. Fluorescent intensity of cells associated with LY beads was calculated and represented as mean fluorescent intensity (MFI). Each bar represents the mean ± SEM of four independent experiments. Differing letters indicate statistical significance (*p* ≤ 0.05) between means. Experimental groups were compared using a one-way ANOVA, followed by the Tukey test using Prism 6 software (GraphPad Software, La Jolla, CA, USA).

As shown in Figure 3A, the activation of 2.6b^ITAM CYT^/1.1b^WT CYT^ co-expressing cells using LY beads opsonized with 0.32 µg/mL α-HA mAb (plus 2.5 µg/mL IgG1) significantly increased intracellular phosphotyrosine staining levels when compared with the same cells activated using LY beads opsonized with 2.5 µg/mL α-FLAG (plus 0.32 µg/mL IgG1); 2897 MFI vs. 185 MFI, respectively. This indicates that the activation of 2.6b^ITAM CYT^ for 15 min triggers intracellular phosphotyrosine-based signaling events while 1.1b^WT CYT^ crosslinking does not. However, when 2.6b^ITAM CYT^/1.1b^WT CYT^ cells were activated using LY beads co-opsonized with 0.32 µg/mL α-HA mAb and 2.5 µg/mL α-FLAG mAb, the MFI value was 931 (Figure 3A). This ~70% reduction (i.e., 2897 vs. 931 MFI) in phosphotyrosine staining intensity shows that co-crosslinking of 2.6b^ITAM CYT^ with 1.1b^WT CYT^ significantly inhibits 2.6b^ITAM CYT^-mediated intracellular signaling events. This may also suggest that co-crosslinking of 1.1b^WT CYT^ with 2.6b^ITAM CYT^ is required to initiate the inhibitory cross-talk actions of IpLITR 1.1b. Finally, as shown in Figure 3B, the tyrosine deficient 1.1b^6YF CYT^ mutant did not reduce phosphotyrosine staining when co-crosslinked with 2.6b^ITAM CYT^, suggesting that an intact tyrosine-containing CYT region is required to mediate the cross-inhibition of 2.6b^ITAM CYT^-mediated intracellular phosphotyrosine signaling.

### 2.4. Examination of IpLITR-Mediated Tyrosine Phosphorylation Staining at Phagocytic Cups

**Figure 4:**
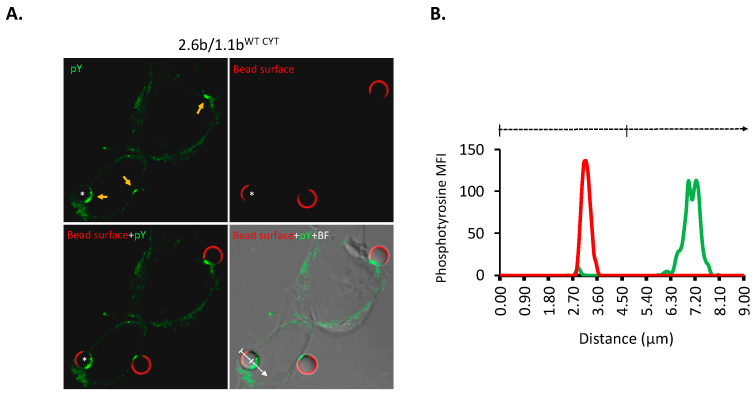

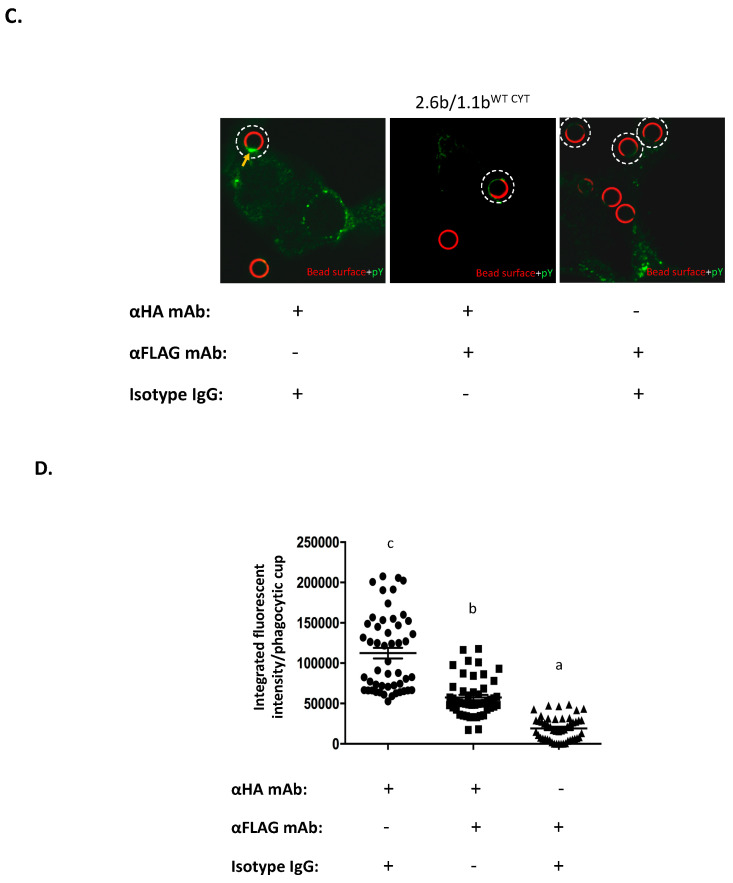
Confocal microscopic analysis of phosphotyrosine staining levels within phagocytic cups. 2.6b^ITAM CYT^/1.1b^WT CYT^ co-expressing AD293 cells (**A**) were grown on coverslips and incubated with 4.5 µm non-fluorescent (NF) beads opsonized with α-HA mAb and mouse isotype IgG1. After 8 min of incubation at 37 °C, cells were fixed with 4% PFA for 10 min and non-phagocytosed beads were stained using Alexa 647-conjugated goat-α-mouse secondary pAb (red). Cells were then permeabilized and specifically stained for intracellular phosphotyrosine molecules by first incubating them with a rabbit α-phosphotyrosine mAb and then with a secondary goat-α-rabbit pAb conjugated to Alexa 488 (green). Z-stack images were obtained at a magnification of 63X using a Zeiss LSM 710 scanning confocal microscope. Representative images from z-stack acquisitions are shown as images with phosphotyrosine (green) staining, surface-exposed bead (red) staining, and merged-fluorescence images or brightfield-fluorescence merged images. Yellow arrowheads show the positions of representative phagocytic cups and the target bead of interest is indicated by an asterisk (*). (**B**) Qualitative analysis of bead and phosphotyrosine molecule staining intensities was performed using ImageJ software (NIH, Bathesda, Maryland, DC, USA) by calculating the MFI (y-axis) of the bead (red line) and phosphotyrosine molecule staining (green line) across the dash arrowed line. (**C**) To further quantify the phosphotyrosine molecules recruited to phagocytic cups under different activation conditions, 2.6b^ITAM CYT^/1.1b^WT CYT^ co-expressing cells were incubated with NF beads opsonized with indicated mAbs (’+’ and ‘−’ indicate the presence and absence of corresponding mAbs, respectively). For analyses, a region of interest (ROI, indicated by dashed circles) was first drawn that includes phagocytic cups so phosphotyrosine signal intensities in this area could be calculated. As summarized in (**D**), at least 50 phagocytic cups from three independent experiments were pooled and the data is represented as mean integrated fluorescent intensity ± SEM. Differing letters indicate statistical significance (*p* ≤ 0.05) between means. Experimental groups were compared using a one-way ANOVA, followed by the Tukey test using Prism 6 software (GraphPad Software, La Jolla, CA, USA).

To examine IpLITR-mediated signaling events at the plasma membrane–target interface (i.e., phagocytic cups) using IpLITR co-expressing AD293 cells, a confocal microscopy-based phagocytosis assay was performed [23,26]. Specifically, α-HA mAb (0.32 µg/mL) opsonized non-fluorescent (NF) 4.5 µm beads were incubated with 2.6b^ITAM CYT^/1.1b^WT CYT^ co-expressing AD293 cells for 8 min at 37 °C. The cells were subsequently stained to detect intracellular phosphotyrosine molecules using a FITC (green)-conjugated rabbit mAb. The samples were also co-stained with an α-mouse IgG mAb conjugated with Cy5 (red) to detect any extracellular exposed surfaces of the NF beads. Intracelluar compartments such as phagosomes as well as plasma membrane-bead contact sites (i.e., phagocytic synapses) at the cell surface are inaccessible to antibody staining. Therefore, regions of the beads that are intimately associated with the plasma membrane during target capture and engulfment processes are not detectable using Cy5 antibody staining (i.e., red) and completely phagocytosed beads are also not stainable using this procedure. Figure 4A shows representative panels of a typical staining pattern using this approach. Notably, as indicated by the yellow arrows, bright green staining is visible at the bead positions devoid of Cy5 staining, suggesting an accumulation of phosphorylated signaling molecules specifically at bead–plasma membrane interfaces. A staining intensity histogram (Figure 4B) records the variable MFIs of Cy5 (bead surface) and FITC (phosphotyrosine) staining across a representative NF bead (marked with an asterisk) along the dashed arrow line. This clearly shows the reciprocal staining patterns for Cy5 and FITC, and nicely demonstrates that the accumulation of phosphorylated molecules occurs positionally within phagocytic cups. To quantify the signal intensities of phosphotyrosine molecules recruited to phagocytic cups, a region of interest (ROI) was established to include representative target beads and phagocytic synapses for the purpose of calculating signal intensities using multiple events (i.e., dashed circles in Figure 4C). For consistency, the size of the ROI is identical for all images analyzed. As shown in Figure 4C and quantified in Figure 4D, the activation of the 2.6b^ITAM CYT^/1.1b^WT CYT^ cells with 2.5 µg/mL of α-FLAG mAb only opsonized NF beads (and 0.32 µg/mL of IgG1) failed to initiate phosphotyrosine signaling (Figure 4C; right panel) within phagocytic cups; FITC MFI = 19,103 ± 2077 within the ROI (Figure 4D). In comparison, 2.6b^ITAM CYT^/1.1b^WT CYT^ cells incubated with 0.32 µg/mL of α-HA mAb only opsonized NF beads (and 2.5 µg/mL of IgG1) triggered a significant increase in FITC staining at the representative bead–cell interface shown (Figure 4C; yellow arrow, left panel), with an overall quantified FITC MFI of 112,488 ± 6717 within the ROIs (Figure 4D). As observed with the phospho-flow assay described earlier (see methods Section 4.3 for details), co-crosslinking of 2.6b^ITAM CYT^ with 1.1b^WT CYT^ suppressed the phosphotyrosine activity induced by 2.6b^ITAM CYT^ crosslinking alone; FITC MFI = 57,427 ± 3351 (Figure 4D), representing an inhibition of the 2.6b^ITAM CYT^-induced tyrosine phosphorylation response by ~50%.

### 2.5. Examination of the Selective Recruitments of Csk and pSHP-2 Molecules to 1.1b^CYT^ Constructs Using Confocal Microscopy

**Figure 5:**
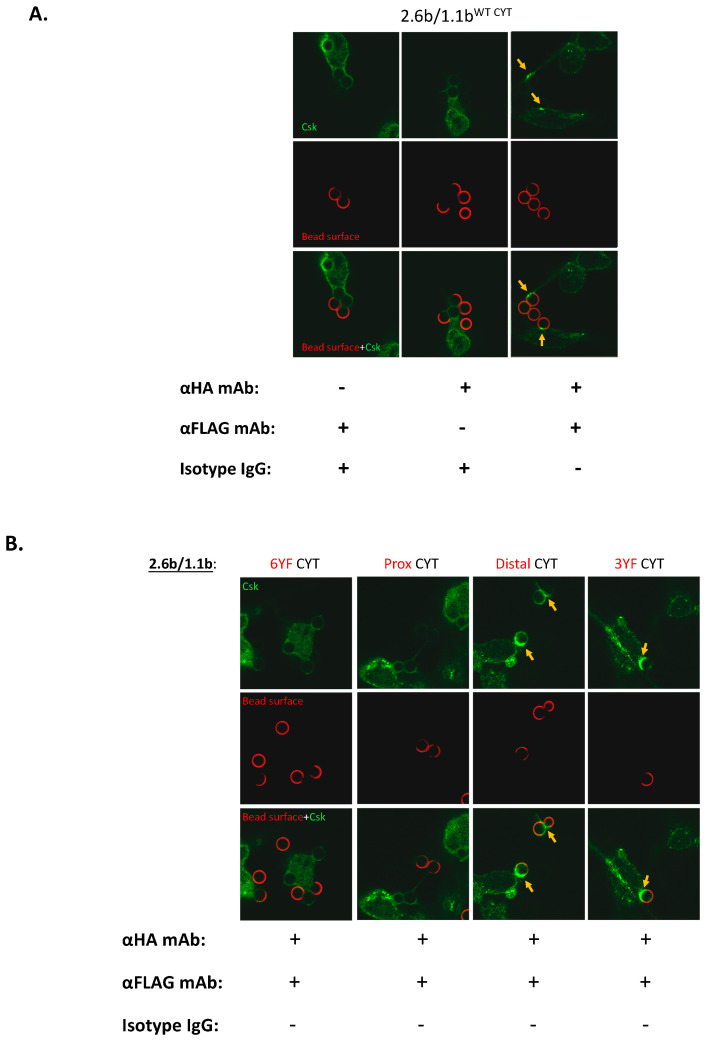

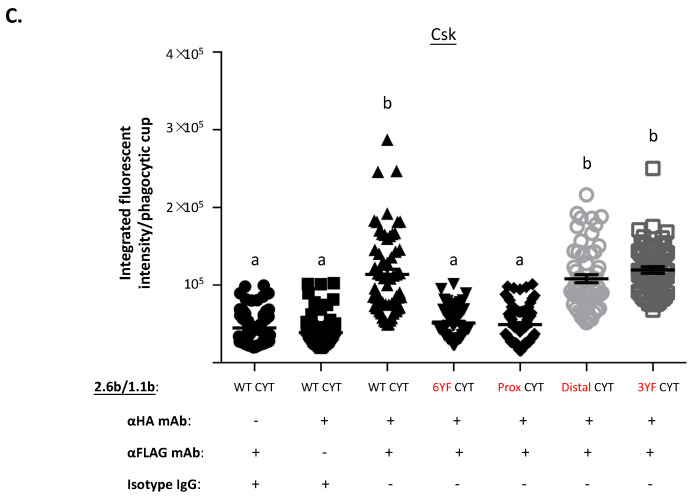
Confocal microscopic analysis of Csk recruitment to phagocytic cups following co-crosslinking of various IpLITR constructs. 2.6b^ITAM CYT^/1.1b^WT CYT^ co-expressing AD293 cells (2 × 10^5^) (**A**) or AD293 cells co-expressing 2.6b^ITAM CYT^ with various mutant 1.1b^CYT^ constructs (**B**) were incubated at 37 °C with 4.5 µm non-fluorescent (NF) beads opsonized with the indicated mAbs and/or isotype IgG1 (’+’ and ‘−’ indicate the presence and absence of corresponding antibodies, respectively). After 8 min, cells were fixed with 4% PFA and non-phagocytosed beads were stained using Alexa 647-conjugated (red) goat-α-mouse secondary pAb. After the bead staining step, the cells were permeabilized and stained for Csk by incubating with a rabbit α-Csk mAb and then a secondary goat-α-rabbit pAb conjugated to Alexa 488 (green). Representative images from z-stack acquisitions are shown with Csk (top panels; green) staining, surface-exposed bead (middle panels; red) staining and merged-fluorescence images (bottom panels). Yellow arrowheads show positions of representative phagocytic cups and the accumulation of Csk staining at locations where extracellular bead staining is absent. (**C**) Quantitative analyses of Csk staining intensities at phagocytic cups were performed using ImageJ software. At least 50 phagocytic cups from three independent experiments were analyzed and the data is represented as mean integrated fluorescent intensity ± SEM. Differing letters indicate statistical significance (*p* ≤ 0.05) between means. Experimental groups were compared using a one-way ANOVA, followed by the Tukey test using Prism 6 software (GraphPad Software, La Jolla, CA, USA).

To determine the identity of candidate effector molecules potentially required for the IpLITR-mediated cross-talk inhibition of the phagocytic response, quantified confocal imaging of Csk and pSHP-2 at the phagocytic synapses were performed. These experiments were similar to those described in the previous section but with an expanded repertoire of constructs tested. In the first set of experiments, the positional recruitment of Csk to phagocytic cups was assessed using 4.5 µm NF beads that were co-opsonized with the mAbs listed below the image panel in Figure 5A (details of the antibody opsonization protocol are described in the Methods Section 4.4). Using the 2.6b^ITAM CYT^/1.1b^WT CYT^ co-expressing AD293 cells, incubation with α-HA and α-FLAG mAb co-opsonized beads, but not α-HA or α-FLAG mAb only opsonized beads, resulted in Csk staining at phagocytic cups (Figure 5A; yellow arrows, right panels). This suggests that co-crosslinking of 1.1b^WT CYT^ with 2.6b^ITAM CYT^ is required to facilitate Csk recruitment to the former construct. Next, using this imaging platform, we then showed that the 1.1b^WT CYT^-mediated recruitment of Csk requires an intact proximal CYT region containing tyrosine 453 (Figure 5B; yellow arrows in the Distal CYT and 3YF CYT imaging panels) when co-crosslinked with 2.6b^ITAM CYT^. Verification of these imaging results was obtained by quantifying the integrated fluorescent intensity/phagocytic cup of Csk recruitment as shown in Figure 5C.

**Figure 6:**
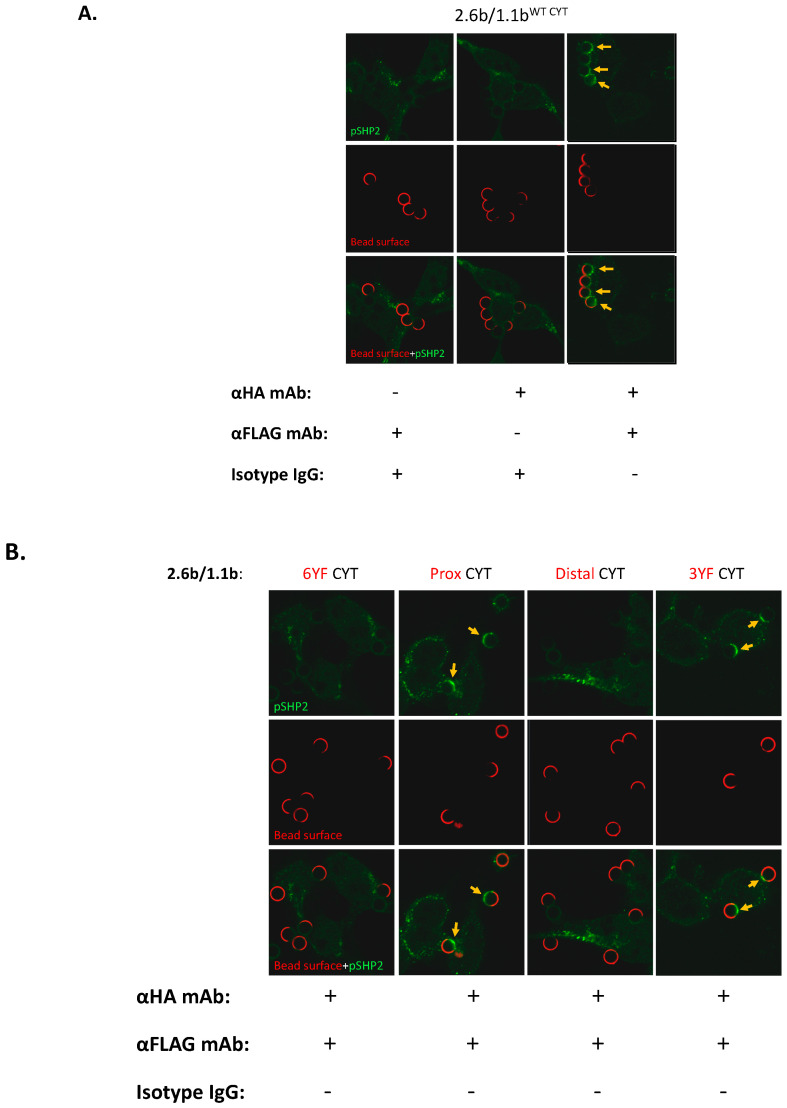

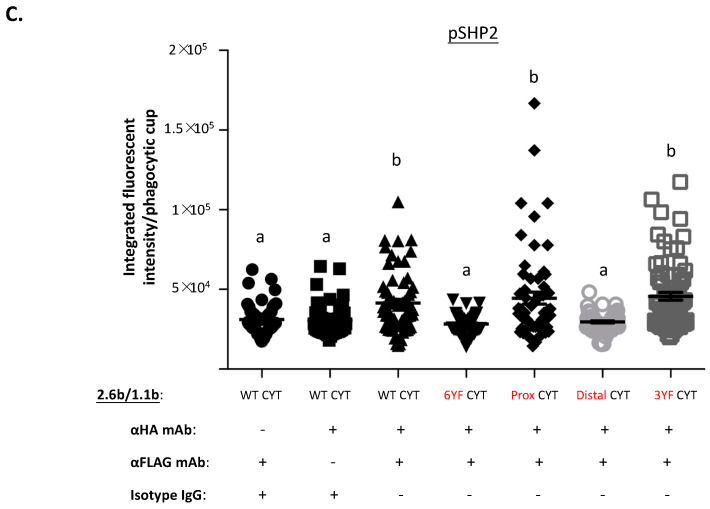
Confocal microscopic analysis of pSHP-2 recruitment to phagocytic cups following co-crosslinking of various IpLITR constructs. 2.6b^ITAM CYT^/1.1b^WT CYT^ co-expressing AD293 cells (2 × 10^5^) (**A**) or AD293 cells co-expressing 2.6b^ITAM CYT^ with various mutant 1.1b ^CYT^ constructs (**B**) were incubated at 37 °C with 4.5 µm non-fluorescent (NF) beads opsonized with the indicated mAbs and/or isotype IgG1 (’+’ and ‘−’ indicate the presence and absence of corresponding antibodies, respectively). After 8 min, cells were fixed with 4% PFA and non-phagocytosed beads were stained using Alexa 647-conjugated (red) goat-α-mouse secondary pAb. After the bead staining step, cells were permeabilized and stained for pSHP-2 by incubating with a rabbit α-pSHP-2 mAb and then a secondary goat-α-rabbit pAb conjugated to Alexa 488 (green). Representative images from z-stack acquisitions are shown with Csk (top panels; green) staining, surface-exposed bead (middle panels; red) staining and merged-fluorescence images (bottom panels). Yellow arrowheads show positions of representative phagocytic cups and the accumulation of pSHP-2 staining at locations where extracellular bead staining is absent. (**C**) Quantitative analyses of pSHP-2 staining intensities at phagocytic cups were performed using ImageJ software. At least 50 phagocytic cups from three independent experiments were analyzed and the data is represented as mean integrated fluorescent intensity ± SEM. Differing letters indicate statistical significance (*p* ≤ 0.05) between means. Experimental groups were compared using a one-way ANOVA, followed by the Tukey test using Prism 6 software (GraphPad Software, La Jolla, CA, USA).

We also examined the recruitment of pSHP-2 in 2.6b^ITAM CYT^/1.1b^WT CYT^ co-expressing cells, again using the mAb-opsonized NF beads listed below the imaging panel in Figure 6A (details of the antibody opsonization protocol are described in the Methods Section 4.4). As observed for Csk, the recruitment of pSHP-2 to the phagocytic cups required the co-engagement of both receptors on 2.6b^ITAM CYT^/1.1b^WT CYT co^-expressing AD293 cells (Figure 6A; yellow arrows, right panel). However, in contrast to Csk recruitment, pSHP-2 required the intact distal CYT region of IpLITR 1.1b, containing tyrosines 477 and 499 which are both embedded within ITIMs (Figure 6B; yellow arrows in the Prox CYT and 3YF CYT staining panels). The quantification of the selective recruitment of pSHP-2 to the 1.1b^WT CYT^, 1.1b^Prox CYT^, and 1.1b^3YF CYT^ constructs when co-crosslinked with 2.6b^ITAM CYT^ is shown in Figure 6C.

### 2.6. Co-Immunoprecipitation-Based Examination of the Selective Recruitments of Csk and pSHP-2 to 1.1b^CYT^ Constructs during Co-Crosslinking with 2.6b^ITAM CYT^

**Figure 7:**
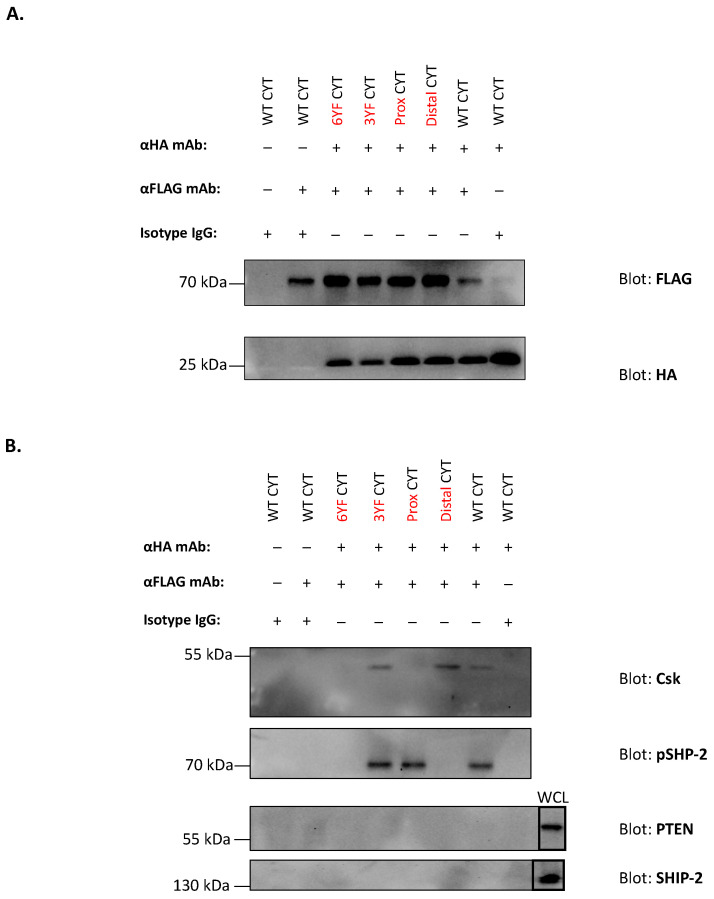
Co-immunoprecipitation of the selective recruitment of Csk and pSHP-2 to various 1.1b^WT CYT^ constructs following co-crosslinking with 2.6b^ITAM CYT^. AD293 cells (3 × 10^5^) co-expressing 2.6b^ITAM CYT^ and the various 1.1b^CYT^ constructs were incubated at 37 °C for 8 min with 3 µm magnetic beads (3 × 10^6^) opsonized with the indicated mAbs and/or isotype IgG1 (’+’ and ‘−’ indicate the presence and absence of corresponding antibodies on beads, respectively). Cells were immediately lysed on ice for 30 min and then magnetic beads were separated, washed three times, and eluted before separating bead-bound proteins using SDS-PAGE. Separated proteins were transferred to nitrocellulose membranes and then probed with α-FLAG and α-HA mAbs (**A**) to verify successful pull-down of the various 1.1b^CYT^ constructs (top membrane) and 2.6b^ITAM CYT^ (bottom membrane). Co-immunoprecipitation of potential effector molecules (**B**) was further examined by probing membranes with α-Csk, α-pSHP-2, α-PTEN, and α-SHIP2 mAbs. For the bottom two panels in (**B**), a whole cell lysate (WCL) sample is included to show that these molecules are present in AD293 lysates. Blots shown are representative examples of three independent experiments.

To support the data obtained from confocal imaging studies, 2.6b^ITAM CYT^ and the various 1.1b^CYT^ constructs (i.e., 1.1b^WT CYT^, 1.1b^6YF CYT^, 1.1b^Prox CYT^, 1.1b^Distal CYT^, and 1.1b^3YF CYT^) were co-crosslinked on AD293 cells using magnetic beads co-opsonized with the α-HA and α-FLAG mAbs. As controls, 2.6b^ITAM CYT^/1.1b^CYT^ construct co-expressing AD293 cells, were incubated with magnetic beads opsonized with α-HA mAb, α-FLAG mAb, or isotype IgG1 alone. After 8 min of incubation at 37 °C with the various antibody-coated beads, the cells were lysed, and magnetic beads were magnetically isolated. Immunoprecipitated proteins were then detected by immunoblotting. Nitrocellulose membranes were probed with α-HA or α-FLAG mAbs to first verify the successful pull-down of the N-terminal HA-tagged 2.6b^ITAM CYT^ or the FLAG-tagged IpLITR1.1b^CYT^ constructs, respectively (Figure 7A). Immunoreactive bands corresponding to 2.6b^ITAM CYT^ (~26 kDa) and 1.1b^CYT^ (~72 kDa) constructs were present using co-opsonized magnetic beads, whereas only 2.6b^ITAM CYT^ or 1.1b^CYT^ constructs were observed using α-HA or α-FLAG mAb opsonized beads, respectively. Notably, no bands were observed when the cells were incubated with isotype IgG1-opsonized magnetic beads (Figure 7A). To validate the selective recruitment of Csk and pSHP-2 molecules to the CYT regions of the various 1.1b^CYT^ constructs, nitrocellulose membranes were also probed using mAbs specific for Csk and pSHP-2 proteins. Immunoreactive bands corresponding to Csk (at ~50 kDa) were only detected after co-crosslinking of 2.6b^ITAM CYT^ with the 1.1b^CYT^ constructs that contained tyrosine 453 (i.e., 1.1b^WT CYT^, 1.1b^Distal CYT^ and 1.1b^3YF CYT^; Figure 7B). In comparison, pSHP-2 bands (at ~70 kDa) were observed after co-crosslinking 2.6b^ITAM CYT^ with the 1.1b^CYT^ constructs containing intact ITIMs located at tyrosines 477 and 499 (i.e., 1.1b^WT CYT^, 1.1b^Prox CYT^ and 1.1b^3YF CYT^; Figure 7B). Of note, independent IpLITR triggering or co-crosslinking of 2.6b^ITAM CYT^ with 1.1b^6YF CYT^, resulted in the pull-down of each IpLITR construct without the associated Csk or pSHP-2 molecules (Figure 7B). Recruitment of other candidate inhibitory effector molecules involved in lipid signaling were also examined; however, no immunoreactive bands corresponding to phosphatase and tensin homolog (PTEN) nor SH2 domain-containing inositol 5′-phosphatase 2 (SHIP-2) were observed even though these signaling molecules were present in the whole cell lysates (Figure 7B) of IpLITR co-expressing AD293 cells.

### 2.7. Inhibition of pErk1/2 Activation Occurs during IpLITR 1.1b-Mediated Cross-Talk Inhibition

**Figure 8:**
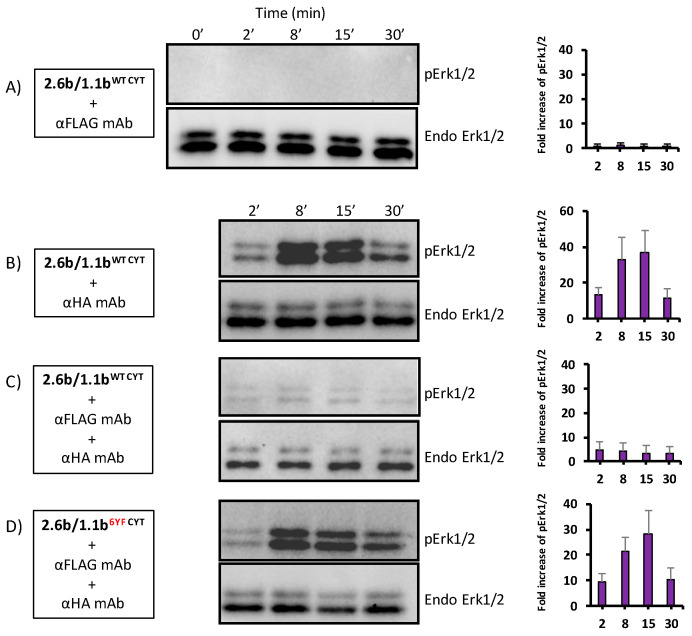

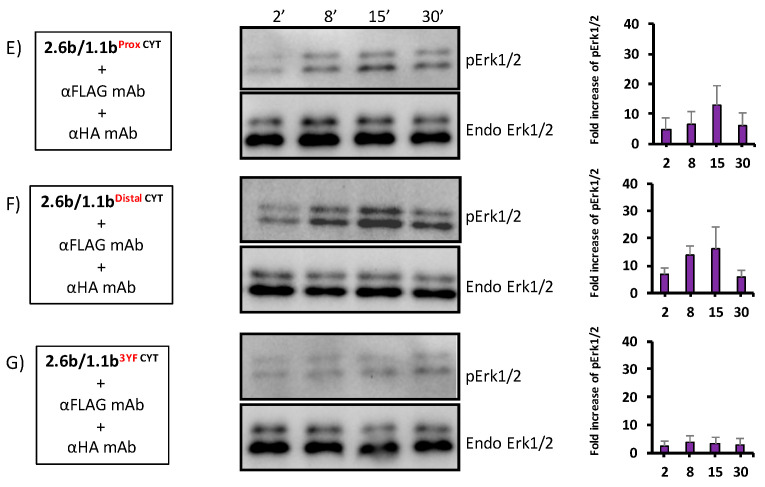
Examination of Erk1/2 activation in IpLITR-activated AD293 cells. 2.6b^ITAM CYT^/1.1b^WT CYT^ co-expressing AD293 cells (3 × 10^5^) were incubated with 4.5 µm YG beads (9 × 10^5^) opsonized with α-FLAG or α-HA mAbs to crosslink 1.1b^WT CYT^ (**A**) or 2.6b^ITAM CYT^ (**B**) constructs for the indicated time points at 37 °C, respectively. Cell lysates were then blotted with either α-pErk1/2 mAb (A, B; top) or α-Erk1/2 mAb (endogenous control; A, B; bottom). In co-crosslinking groups, the same experimental procedures were used, but the 4.5 µm YG beads were co-opsonized with α-FLAG and α-HA mAbs to co-crosslink 2.6b^ITAM CYT^ with the various 1.1b^CYT^ constructs (**C**–**G**). Band intensity values were obtained by densitometry using ImageJ software. Relative changes in pErk1/2 levels are reported as fold induction values relative to untreated 2.6b^ITAM CYT^/1.1b^WT CYT^ stable cells (i.e., 0 min), which is set to 1.0. pErk1/2 band intensity levels were corrected for endogenous molecule levels using the following equation: pErk1/2 densitometry value for the time point / endo-Erk value for the time point. This corrected value was then divided by the value obtained at 0 min (i.e., unstimulated cells). The results are representative of three independent experiments and band intensity values represented as mean ± SEM.

Having established that 1.1b^WT CYT^-mediated cross-inhibition of phagocytosis likely operates through the selective recruitment of Csk and pSHP-2 molecules, we next examined a candidate signaling pathway that may be affected during IpLITR-mediated cross-talk inhibition. Since phagocytic signaling is a dynamic spatiotemporal process, we measured downstream Erk1/2 phosphorylation over a 30 min time course. The independent engagement of 1.1b^WT CYT^ for 2, 8, 15, and 30 min did not induce Erk1/2 phosphorylation (Figure 8; panel A). In contrast, the activation of 2.6b^ITAM CYT^ or co-crosslinking of 2.6b^ITAM CYT^ with 1.1b^6YF CYT^ triggered Erk1/2 phosphorylation as early as 2 min following receptor activation (~10 fold increase relative to 0 min), which peaked at 15 min (~30 fold increase) and then subsided by 30 min (~10 fold increase) post-stimulation (Figure 8; panels B and D). A clear reduction of Erk1/2 phosphorylation levels in AD293 cells was observed after co-crosslinking 2.6b^ITAM CYT^ with 1.1b^WT CYT^ (Figure 8; panel C). To further investigate which tyrosine motifs are required to down-regulate Erk1/2 phosphorylation, the phosphorylation state of Erk1/2 was examined after co-crosslinking 2.6b^ITAM CYT^ with various tyrosine mutant 1.1b^CYT^ constructs. These experiments showed that the 1.1b^Prox CYT^ construct diminished but did not abolish Erk1/2 activation (Figure 8; panel E) when co-crosslinked with 2.6b^ITAM CYT^, indicating that the proximal CYT region of IpLITR 1.1b is required for the optimal inhibition of this pathway and that the ITIM-containing distal region alone is not sufficient for the maximal reduction of Erk1/2 activation. Surprisingly, mutation of the distal CYT region tyrosines shows that a CYT proximal-dependent inhibition of the Erk1/2 pathway, although modest, may be operating (Figure 8, panel F). Finally, the CYT construct containing only tyrosines 453, 477, and 499 shows that a cooperative IpLITR 1.1b CYT proximal and distal region mechanism likely achieves maximal inhibition of the Erk1/2 pathway (Figure 8, panel G), similar to the overall inhibition observed when 2.6b^ITAM CYT^ was co-crosslinked with the non-mutated 1.1b^WT CYT^ construct (Figure 8, compare panels C and G).

## 3. Discussion

In this study, we have showed that 1.1b^WT CYT^ has potent cross-talk inhibitory effects on 2.6b^ITAM CYT^-mediated phagocytosis through the coordinated recruitments of select inhibitory molecules. Of note, our results relied on the heterologous overexpression of IpLITRs in a surrogate, but well-characterized mammalian cell line; as such, the results described here should be considerate of this fact. However, this general approach has been successfully used as a model for the functional characterization of other immunoregulatory receptor-types, including those that regulate mammalian immunity [28,29,30,31,32]. For example, a chimeric human FcγRIIA receptor containing the extracellular domain of FcγRI and the transmembrane and cytoplasmic region of FcγRIIA was co-expressed with FcγRIIB to alleviate the complexities associated with the fact that several different FcγR classes are expressed in hematopoietic cells combined with the lack of specific mAbs targeting individual FcγR isoforms [10]. Therefore, co-expression of two FcγRII isoforms of interest in a heterologous system provides a reductionist approach that can help eliminate confounding factors (e.g., activation of other FcγR isoforms), thus allowing for specific investigations of the effects of cross-talk interactions between two different FcγR-types. As described our study, we used a similar approach, whereby 2.6b^ITAM CYT^ and 1.1b^CYT^ constructs were first differentially tagged with the N-terminal HA and FLAG epitopes, respectively, and then stably co-expressed in AD293 cells. This co-expression system allowed us to specifically activate each IpLITR construct independently or co-engage them using commercially available epitope-specific mAbs directionally opsonized on microbeads. To validate the precision of this system, IpLITR co-expressing AD293 cells were incubated with microbeads opsonized with pre-calibrated concentrations of α-HA and/or α-FLAG-specific mAbs. Our results showed that independent crosslinking of 2.6b^ITAM CYT^ caused a robust phagocytic response, whereas only target binding activity was observed following the specific triggering of 1.1b^WT CYT^. This is consistent with phenotypes observed in AD293 cells solely expressing 2.6b ^ITAM CYT^ and 1.1b^WT CYT^ [24] and rules out the possibility of cross-reactivity between α-HA and α-FLAG mAbs in these assays. This co-expression system also allowed us to optimize the concentrations of the, mAbs co-opsonized on microbead targets when engaging different IpLITR-types during co-crosslinking experiments. Consequently, the results observed after co-crosslinking the IpLITR-types are unlikely due to the disproportionate activation of one IpLITR-type versus the other, but rather show functional differences due to the compositions of the co-engaged receptors (i.e., co-engagements of 2.6b^ITAM CYT^ and 1.1b^WT CYT^ vs. 2.6b^ITAM CYT^ and 1.1b^6YF CYT^). 

The initiation of phagocytosis is associated with increased tyrosine phosphorylation of intracellular signaling proteins, which is counter-balanced by the reduction of phosphotyrosine signaling events by phosphatases (e.g., SHPs) that specifically target kinases (e.g., Syk) responsible for activating the phagocytic machinery and other cellular signaling pathways [29,30,33]. Our results show that the inhibition of 2.6b^ITAM CYT^-mediated phagocytosis is also associated with a significant reduction in phosphotyrosine signals after co-crosslinking with 1.1b^WT CYT^. Interestingly, this reduced phosphotyrosine level is still significantly higher than the basal level of tyrosine phosphorylation observed when 1.1b^WT CYT^ is independently crosslinked, indicating that a low level of intracellular tyrosine phosphorylation remains after receptor co-crosslinking. It is possible that 2.6b^ITAM CYT^-dependent activating signals are not completely counter-balanced/reduced by phosphatases recruited to 1.1b^WT CYT^ and this may account for the residual phagocytic activity observed after co-crosslinking 2.6b^ITAM CYT^ with 1.1b^WT CYT^. Alternatively, inhibitory molecules recruited to 1.1b^WT CYT^ during receptor cross-talk may also be targeted for phosphorylation as we observed these events only during the 2.6b^ITAM CYT^-mediated activation of 1.1b^WT CYT^-recruited signaling molecules (i.e., SHP-2). Regulatory tyrosine phosphorylation has been reported to increase the enzymatic activity of phosphatases, such as SHP-1 and SHP-2 [33,34,35,36,37,38,39]. Of note, the independent engagement of 1.1b^WT CYT^ showed only basal levels of phosphorylation, similar to the levels observed when the 1.1b^6YF CYT^ construct was triggered. This suggests that triggering of 1.1b^WT CYT^ alone is not sufficient to initiate phosphotyrosine-dependent inhibitory signals from this receptor and raises the interesting possibility that the co-crosslinking of 1.1b^WT CYT^ with 2.6b^ITAM CYT^ is likely a required prerequisite for the phosphorylation and ‘activation’ of the inhibitory abilities of 1.1b^WT CYT^. Indeed, recruitment of Csk and pSHP-2 to 1.1b^WT CYT^ was only observed after co-crosslinking this receptor with the with 2.6b^ITAM CYT^ construct. This apparent trans-phosphorylation mechanism of inhibitory receptors when co-engaged with activating counterparts has been reported by others and as shown in our study, it appears to be an important immunoregulatory cross-talk mechanism mediated by IpLITRs [40,41]. For example, independent engagements of FcγRIIB failed to phosphorylate tyrosines within its CYT region; whereas after co-crosslinking, Src family kinases (SFKs; e.g., Lyn) recruited to the activating FcϵRI trans-phosphorylated the ITIMs of FcγRIIB which consequently facilitated the recruitment of SHIPs required for the cross-inhibition of FcϵRI-mediated degranulation in mast cells [40]. A similar mechanism could help explain the activation of 1.1b^WT CYT^ as it likely translocates into lipid rafts where SFKs are resident after co-crosslinking with its activating counterparts. In return, phosphorylated 1.1b^WT CYT^ generates inhibitory signals to cross-inhibit signaling events downstream of 2.6b^ITAM CYT^ activation. In this scenario, 2.6b^ITAM CYT^ is not a passive target of inhibitory effectors but instead actively participates in its own inhibition by facilitating the phosphorylation of 1.1b^WT CYT^ and the subsequent recruitment and activation of inhibitory phosphatases. Overall, this represents an intrinsic negative feedback loop for controlling the ITAM-mediated induction of the phagocytic response by vertebrate immunoregulatory receptors including FcRs and IpLITRs. 

**Figure 9:**
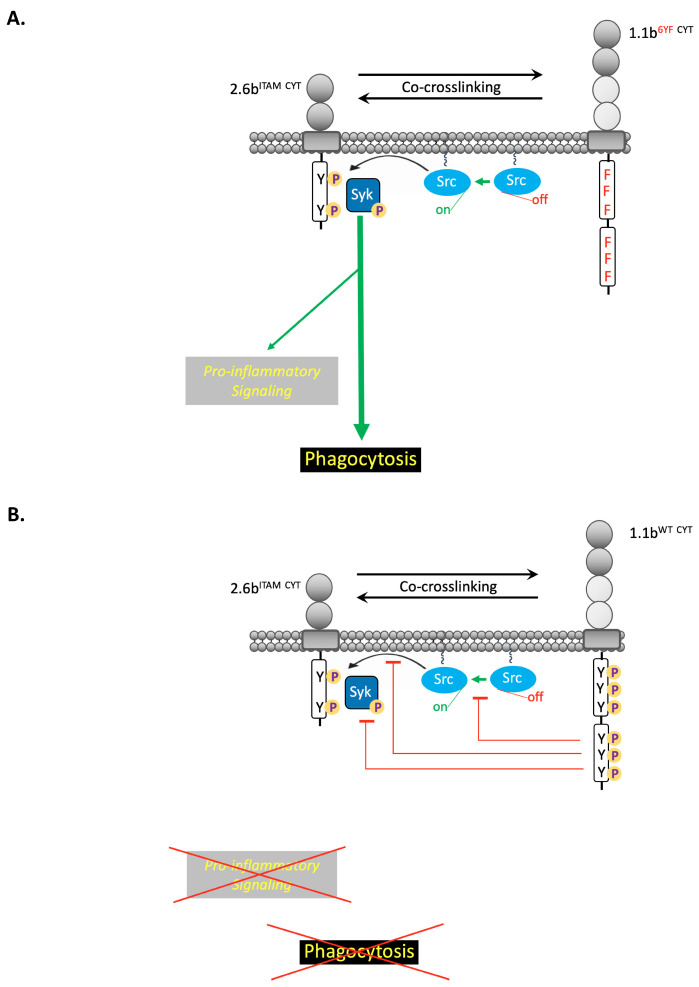

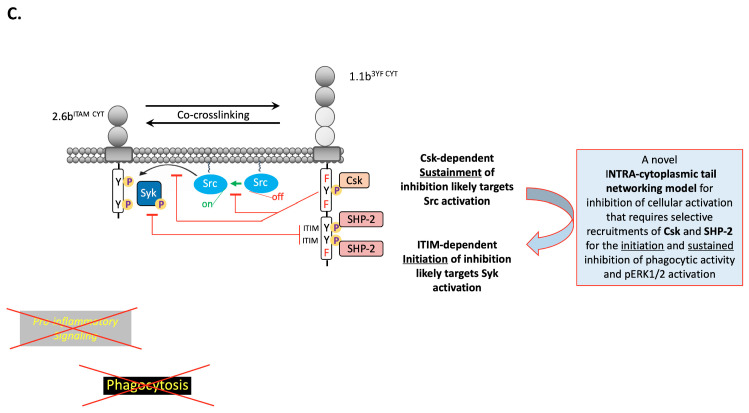
Hypothesized mechanism for the 1.1b^WT CYT^-mediated cross-inhibition of ITAM-driven phagocytosis and cellular activation. Co-crosslinking of 2.6b^ITAM CYT^ with 1.1b^6YF CYT^ (**A**) when compared to co-crosslinking of 2.6b^ITAM CYT^ with 1.1b^WT CYT^ (**B**) provides the basis for the proposal of a novel intra-cytoplasmic tail networking model (**C**) that requires selective recruitment of Csk and SHP-2 by IpLITR 1.1b for initiation and sustainment of cross-inhibition as detailed in the discussion.

The inhibitory roles of 1.1b^WT CYT^ were originally characterized in transfected NK cells, and the first functional report showed that 1.1b^WT CYT^-mediated inhibition of NK cell-mediated cytotoxicity likely operates through the recruitment of phosphatases at ITIMs located within its membrane distal CYT region [18]. Further studies also revealed an ITIM-independent mechanism of 1.1b^WT CYT^-mediated inhibition of NK cell killing responses. Specifically, the membrane proximal CYT region of IpLITR 1.1b was capable of independently abrogating NK cell cytotoxicity and subsequent biochemical analysis identified a Csk-binding motif (i.e., at tyrosine 453) responsible for this inhibitory activity [19]. Contrary to the observation that membrane proximal and distal CYT segments of 1.1b^WT CYT^ independently inhibited NK cell cytotoxicity, tyrosine-based motifs (i.e., located at tyrosine 453, 477, and 499) were minimally required to initiate and sustain the inhibition of 2.6b^ITAM CYT^-mediated phagocytosis in the present study. Combined with the observations obtained from biochemical and imaging assays, we propose a model in which the proximal and distal CYT regions of 1.1b^WT CYT^ work in concert to initiate and then sustain inhibitory signals through the selective recruitment of Csk and SHP-2 following co-crosslinking with 2.6b^ITAM CYT^. Consistent with our previous studies in which 2.6b^ITAM CYT^ was independently engaged [17], the robust induction of phagocytosis and pro-inflammatory signals (i.e., Erk1/2 phosphorylation) were observed after co-crosslinking of 2.6b^ITAM CYT^ with the mutant construct 1.1b^6YF CYT^. These canonical ITAM-dependent signaling events likely require the participation of SFKs to phosphorylate the tandem ITAM tyrosines, recruit Syk, and phosphorylate this enzyme to propagate downstream signaling cascades that promote phagocytosis (Figure 9A). However, 2.6b^ITAM CYT^-mediated phagocytosis was suppressed after co-crosslinking 2.6b^ITAM CYT^ with the functionally capable 1.1b^WT CYT^ construct, and this response is likely due to the recruitment of inhibitory molecules to the 2.6b^ITAM CYT^-1.1b^WT CYT^ complex that suppresses the activities of Syk and SFKs (Figure 9B). Indeed, two specific inhibitory molecules, Csk and SHP-2, were co-immunoprecipitated and recruited to phagocytic cups after co-crosslinking of 2.6b^ITAM CYT^ and 1.1b^WT CYT^. Presumably, Csk and SHP-2 molecules differentially target intracellular substrates but they can also act in concert to exert maximum inhibitory effects on the ITAM-driven phagocytic process. Based on our findings, the recruitment of SHP-2 molecules to the ITIMs located within the distal region of 1.1b^WT CYT^ serve to initiate inhibitory signals by targeting Syk phosphorylation and possibly downstream Syk-initiated signaling (Figure 9C). 

SHP-2 is a ubiquitously expressed phosphatase that inhibits various effector responses by dephosphorylating critical signaling components [42,43,44]. Although specific substrates targeted by SHP-2 in cross-regulating phagocytosis remain to be identified, one possibility is that SHP-2 diminishes the activity of Syk by dephosphorylating its regulatory tyrosines. Syk serves as a critical signaling hub during phagocytosis by phosphorylating and activating various downstream effector molecules, while also supporting the formation of signaling complexes by recruiting SH2 domain-containing molecules to its regulatory tyrosines, which are themselves phosphorylated [45,46,47,48,49]. Specifically, the reduced phosphorylation of Syk tyrosine 346 is responsible for the delayed initiation of phagocytosis in mouse macrophages [50]. This is consistent with the observation in 2.6b^ITAM CYT^/1.1b^Prox CYT^ co-expressing AD293 cells that recruitment of SHP-2 alone significantly inhibits phagocytosis at an early time point (i.e., at 15 min) but fails to sustain the inhibitory effect over time (i.e., at 30 min). Furthermore, the peptide sequence ESP**Y**ADPEE, which flanks regulatory tyrosine 352 (bold) in human Syk is surrounded by acidic amino acids that matches an established SHP-2 binding motif [51]. In addition, this regulatory tyrosine residue is responsible for the binding and activation of phospholipase Cγ1 (PLCγ1) [52]. Therefore, dephosphorylation of this regulatory tyrosine residue by SHP-2 may reduce the activity of PLCγ1, resulting in defective Ras activation, which is also upstream of Erk1/2 phosphorylation [53,54]. Indeed, lower magnitudes of Erk1/2 phosphorylation were observed after co-crosslinking 2.6b^ITAM CYT^ with 1.1b^Prox CYT^ (i.e., SHP-2 recruitment only) compared with 1.1b^Distal CYT^ (i.e., Csk recruitment only) as shown in our biochemical experiments. However, the dephosphorylation of regulatory residues on Syk molecules by SHP-2 fails to sustain the inhibitory effects on phagocytosis at a later time point (i.e., 30 min) as proximal Src family kinase activity may reconstitute the phosphorylation and activation of Syk. Theoretically, this would eventually override inhibitory signals from SHP-2, thus allowing phagocytosis to resume once an activation threshold is established. This also supports the notion that alternative inhibitory mechanisms are required to sustain the inhibition of phagocytosis at later time points. We found that tyrosine 453 within the proximal region of 1.1b^WT CYT^ is indispensable for recruiting Csk and sustaining the inhibitory effects on phagocytosis at 30 min (Figure 9C). Csk is a potent inhibitor of SFKs by specifically phosphorylating a regulatory C-terminal tyrosine, rendering SFKs in an auto-inhibited conformation [55,56]. The loss of SFK activity would prevent the phosphorylation of tyrosines within ITAMs, and also sustain the inhibitory effects of 1.1b^WT CYT^ by reducing further recruitment and subsequent activation of Syk. Therefore, our data supports a novel intracytoplasmic tail networking mechanism via selective recruitment of Csk and SHP-2 to the proximal and distal regions of 1.1b^WT CYT^, respectively (Figure 9C). Specifically, these inhibitory molecules may coordinately suppress proximal signaling components of ITAM signaling (e.g., Syk and SFKs) to both initiate and sustain the inhibition of cellular activation (Figure 9C). Interestingly, although phosphorylation of ITAMs by SFKs precedes the recruitment of Syk, a blockage of SFK activity alone by Csk was not sufficient to initiate an inhibitory effect since 2.6b^ITAM CYT^/1.1b^Distal CYT^ co-expressing cells maintained phagocytic activity following co-crosslinking. In this case, it is likely that SFKs remain active at the phagocytic synapse and continue to phosphorylate tyrosine 453 in the CYT of 1.1b^WT CYT^ to maintain recruitment of Csk molecules. However, without SHP-2-mediated blockage of Syk, this is insufficient for initiating an inhibitory effect, further indicating that intra-cytoplasmic networking and the coordinated recruitment of Csk and SHP-2 are required for optimal cross-talk-mediated inhibition of effector responses.

In an attempt to expand the repertoire of recruited effector molecules involved in fine-tuning phagocytic signals, we also examined two additional inhibitory molecules (i.e., SHIP-2 and PTEN) that have been reported to negatively regulate phagocytosis via the disruption of the PI3K/Akt signaling axis [57,58,59,60]. However, these two molecules were not co-immunoprecipitated with 1.1b^WT CYT^ (Figure 7B) and this may explain our non-published observation that the level of phosphorylated Akt was not affected following IpLITR co-crosslinking. Currently, detailed mechanisms responsible for 1.1b^WT CYT^-mediated cross-inhibition of phagocytosis are far from completely understood and it is unlikely that Csk and SHP-2 are the only inhibitory molecules involved in this process. Future studies to identify potential inhibitory molecules along with their substrates in a more physiologically-relevant system (e.g., fish immune cells) will provide a better understanding of the mechanistic details regarding the cross-inhibitory activities of teleost immunoregulatory receptor-types.

In summary, we have revealed new mechanistic details supporting a model for immunoregulatory receptor-mediated cross-inhibition of the phagocytic response. This represents the first detailed examination of molecular mechanisms underlying teleost receptor-mediated cross-talk during the control of an innate effector response. This study also describes an alternative platform for exploring the dynamic signaling potentials of teleost LITRs that will serve as an important stepping-stone for understanding the in vivo roles of this diverse and complex immunoregulatory receptor family. 

## 4. Materials and Methods 

### 4.1. Generation of AD293 Cell Lines Co-Expressing N-Terminal HA- and FLAG-Tagged IpLITR Constructs

The N-terminal hemagglutinin (HA) epitope-tagged pDisplay-IpLITR 2.6b/IpFcRγ-L (referred to as 2.6b^ITAM CYT^) and pDisplay-IpLITR 1.1b (referred to as 1.1b^WT CYT^) were previously generated as described [17,21,22]. Briefly, 2.6b^ITAM CYT^ is a chimeric receptor that contains the two extracellular Ig-like domains of IpLITR 2.6b (Genbank Accession: ABI23577) fused to the transmembrane (TM) segment and cytoplasmic tail (CYT) region of the ITAM-containing channel catfish adaptor molecule IpFcRγ-L (Genbank Accession: AF543420). The 1.1b^WT CYT^ construct (Genbank Accession: ABI16050) contains four extracellular Ig-like domains, a TM segment and a CYT region containing six tyrosines. The pDisplay-1.1b^WT CYT^ construct was the template for generating various N-terminal FLAG epitope-tagged pCMV-9-1.1b^CYT^ constructs with combinations of mutated tyrosines. Specifically, 1.1b^6YF CYT^ is a construct with all six tyrosines in the CYT of 1.1b^WT CYT^ mutated to phenylalanines (YF); 1.1b^Prox CYT^ has all three tyrosines located in its membrane proximal CYT region (i.e., tyrosine 433/453/463) mutated; 1.1b^Distal CYT^ has all three tyrosines located in its membrane distal CYT region (i.e., tyrosine 477/499/503) mutated, and; 1.1b^3YF CYT^ has tyrosine 453 (i.e., Csk-binding motif) and its two ITIM tyrosines (i.e., tyrosine 477 and 499) intact with the other three CYT region tyrosines all mutated. Mutants were generated using the QuickChange Lightning Site-directed Mutagenesis Kit (Agilent Technologies, Santa Clara, CA, USA) as previously described [21]. The construct sequences were sub-cloned using HindIII/BamHI restriction digest sites into the pCMV-9 eukaryotic expression vector (Sigma-Aldrich, St. Louis, MO, USA), which adds an N-terminal FLAG epitope tag. 

AD293 cells stably expressing HA-tagged 2.6b^ITAM CYT^ were generated previously [24] and then co-transfected with the various pCMV-9-1.1b^CYT^ constructs (i.e., 1.1b^WT CYT^, 1.1b^6YF CYT^, 1.1b^3YF CYT^, 1.1b^Prox CYT^, and 1.1b^Distal CYT^) to generate N-terminal HA-tagged 2.6b^ITAM CYT^/N-terminal FLAG-tagged 1.1b^CYT^ construct co-expressing cell lines. Briefly, 1 µg of CMV-9 plasmid was transfected into 2.6b^ITAM CYT^-expressing AD293 cells cultured in 24-well plates using TurboFect in vitro transfection reagent (Thermo Fisher Scientific), according to the manufacturer’s instructions. Co-transfected cells were then cultured in DMEM (GE Healthcare, Baie d’Urfe, QC, Canada) supplemented with 10% (*v*/*v*) heat-inactivated fetal bovine serum (FBS; GE Healthcare) and 800 µg/mL G418 disulfate salt solution (Sigma-Aldrich, St. Louis, MO, USA) at 37 °C with 5% CO_2_. After 48 h of selection, cells were serially diluted in round bottom 96-well tissue culture plates as described [23]. Individual clones were then screened for co-expression of the epitope-tagged IpLITRs. The clones were harvested as previously described [24], and aliquoted (~1 × 10^5^cells) into 1.5 mL Eppendorf tubes for staining. The cells were centrifuged at 500× *g* for 2 min and cell pellets were gently disrupted after adding 0.1 µg of primary α-HA mAb, α-FLAG mAb or mouse isotype IgG1 (Beckman Coulter, Mississauga, ON, Canada) antibody control diluted in 50 µL of flow cytometry buffer (D-PBS, 0.5% bovine serum albumin, 2mM EDTA, 0.05% NaN_3_). The cells were incubated on ice for 30 min followed by the addition of 1 mL ice-cold flow cytometry buffer, centrifuged at 500× *g* for 2 min, and supernatants were aspirated. Cell pellets were agitated and then 50 µL of flow cytometry buffer containing 0.25 µg of goat anti-mouse IgG (H+L)-PE (Thermo Fisher Scientific, Ottawa, ON, Canada) was added and the staining/washing steps repeated as above. The cells were then analyzed for surface staining using flow cytometry (Beckman Coulter, Mississauga, ON, Canada) as previously described [24]. AD293 cell lines co-expressing 2.6b^ITAM CYT^ and each 1.1b^CYT^ construct were individually verified by co-staining (referred to as 2.6b^ITAM CYT^/1.1b^WT CYT^, 2.6b^ITAM CYT^/1.1b^6YF CYT^, 2.6b^ITAM CYT^/1.1b^Prox CYT^, 2.6b^ITAM CYT^/1.1b^Distal CYT^, and 2.6b^ITAM CYT^/1.1b^3YF CYT^).

### 4.2. Imaging Flow Cytometry-Based Bead-Binding and Phagocytosis Assays

To confirm that each IpLITR construct could be specifically activated in the co-expression AD293 system, HA-tagged 2.6b^ITAM CYT^ and FLAG-tagged 1.1b^WT CYT^ constructs were independently activated using 4.5 µm yellow-green (YG) beads (Polysciences, Inc, Warrington, PA, USA) opsonized with respective mAbs and an imaging flow cytometry-based phagocytosis assay was performed as previously described [24]. Briefly, 4.5 µm YG beads pre-adsorbed with protein A (from *Staphylococcus aureus*; Sigma Aldrich, St. Louis, MO, USA) were co-opsonized with a range of α-HA mAb (i.e., 0.16 µg/mL, 0.32 µg/mL, 0.64 µg/mL, and 1.28 µg/mL) and α-FLAG mAb (i.e., 0.3 µg/mL, 0.64 µg/mL, 2.5 µg/mL, and 5 µg/mL). YG beads were also opsonized with 5 µg/mL mouse isotype IgG1 control. AD293 cells co-expressing 2.6b^ITAM CYT^/1.1b^CYT^ (3 × 10^5^) were then seeded in 24-well tissue culture plates one day prior to experiments. The next day, cell media was aspirated, and cells were incubated in phagocytosis buffer (1:1 mixture of 1 × PBS containing 2 mg/mL BSA and 1X Opti-MEM reduced serum medium; Gibco, Ottawa, ON, Canada) containing 9 × 10^5^ opsonized YG beads. Cell–bead interactions were synchronized by a 1 min 4°C centrifugation at 100× *g*. After a 15 or 30 min incubation at 37 °C, cells were rinsed with ice-cold PBS and then incubated for 30 min with ice-cold phagocytosis buffer containing 2 μg Alexa 647-conjugated rabbit-α-mouse IgG secondary Ab (Thermo Fisher Scientific) to differentially stain extracellularly-exposed bead surfaces. Cells were then rinsed once with ice-cold phagocytosis buffer and harvested using 0.05% trypsin/EDTA (Gibco). After re-suspension, the harvested cells were washed with ice-cold phagocytosis buffer and fixed in 1% paraformaldehyde (PFA, Sigma-Aldrich) prior to analysis using an ImageStream X Mark II instrument (Amnis Corporation). For each sample, 5000 events were collected and data were analyzed using IDEAS^®^ software (Amnis) to resolve two different phenotypes: i) % of cells with surface-bound beads only, and ii) % of cells with at least one phagocytosed bead, as previously described [27].

The optimization of the concentration of mAbs used to co-opsonize the beads was performed using a bead-binding assay. Specifically, 4.5 µm YG beads were opsonized with a range of α-HA or α-FLAG mAb concentrations as described above. To determine the optimal concentration of each mAb to use in the functional assays, YG beads were co-opsonized with α-HA mAbs (0.16–1.28 µg/mL) and isotype IgG1 (0.32–5.0 µg/mL) or α-FLAG mAb (0.32–5.0 µg/mL) and isotype IgG1 (0.16–1.28 µg/mL). Mouse isotype IgG1 was included to ensure that total IgG concentrations were identical on all beads used. For the binding assay, 2.6b^ITAM CYT^/1.1b^WT CYT^ and 2.6b^ITAM CYT^/1.1b^6YF CYT^ co-expressing AD293 cells (3 × 10^5^) were seeded in 24-well tissue culture plates. The next day, the cell media was aspirated, cells were rinsed with ice-cold PBS, and then incubated in an ice-cold phagocytosis buffer on ice for 15 min before the addition of 9 × 10^5^ co-opsonized 4.5 µm YG beads. Cell–bead interactions were synchronized by 1 min 4 °C centrifugation at 100× *g*. After 60 min, cells were rinsed with ice-cold phagocytosis buffer and fixed in 1% PFA prior to analysis using an ImageStream X Mark II instrument. For each sample, 5000 events were collected and data was analyzed using IDEAS^®^ software. 

To investigate the cross-talk potential between IpLITR-types, the different epitope-tagged constructs were co-crosslinked on AD293 cells (i.e., 2.6b^ITAM CYT^/1.1b^WT CYT^, 2.6b^ITAM CYT^/1.1b^6YF CYT^, 2.6b^ITAM CYT^/1.1b^Prox CYT^, 2.6b^ITAM CYT^/1.1b^Distal CYT^, and 2.6b^ITAM CYT^/1.1b^3YF CYT^) using YG beads opsonized with α-HA (0.32 μg/mL) and α-FLAG mAbs (2.5μg/mL) to specifically co-crosslink 2.6b^ITAM CYT^ with the various 1.1b^CYT^ constructs for 15 or 30 min.

### 4.3. Imaging Flow Cytometry-Based Intracellular Phospho-Tyrosine Staining

To determine the intracellular phosphorylation status of cells after co-crosslinking of IpLITR constructs, a phospho-flow assay was performed. Briefly, pre-adsorbed light-yellow (LY, 3.55 μm; Spherotech Inc) latex beads were opsonized with α-HA mAb (0.32 μg/mL) and α-FLAG mAb (2.5 μg/mL). Independent activation of 2.6b^ITAM CYT^ and the various 1.1b^CYT^ constructs was also performed using LY beads opsonized with α-HA mAb (0.32 μg/mL) and mouse isotype IgG1 (2.5 μg/mL) or α-FLAG mAb (2.5 μg/mL) and mouse isotype IgG (0.32 μg/mL) alone. For the phospho-flow assay, 3 × 10^5^ IpLITR co-expressing AD293 cells (i.e., 2.6b^ITAM CYT^/1.1b^WT CYT^ and 2.6b^ITAM CYT^/1.1b^6YF CYT^) were seeded in 24-well tissue culture plates and a pre-warmed (37 °C) phagocytosis buffer containing 9 × 10^5^ opsonized 3.55 μm LY beads was added. Cell–bead interactions were then synchronized by a 1 min 4 °C centrifugation at 100× *g*. After 15 min at 37 °C, the cells were rinsed with ice-cold PBS and fixed for 10 min using 1% PFA. The cells were then washed with PBS and permeabilized using 0.1% saponin (Sigma-Aldrich) diluted in PBS for 5 min at room temperature. Cells were washed with PBS and intracellular phosphotyrosine-containing proteins were detected by staining with an Alexa 488-conjugated α-phosphotyrosine antibody (1:50 dilution in 5% BSA plus 0.1% saponin; Cell Signaling Technology, Danvers, MA, USA). After a 1 h incubation at room temperature, the cells were washed with PBS and re-suspended in 1% PFA prior to analysis using an ImageStream X Mark II instrument. For each sample, 3000 events were collected and phosphotyrosine signals were identified and calculated using the intensity mask feature in the IDEAS^®^ software as described in [27].

### 4.4. Confocal Microscopy-Based Examination of the Recruitment of Intracellular Signaling Molecules during IpLITR-Mediated Cross-Talk Regulation of Phagocytosis

To examine the recruitment of effector molecules to phagocytic cups during IpLITR-mediated regulation of phagocytosis, a modified confocal microscopy-based phagocytosis assay was performed as previously described [20,26]. Specifically, 4.5 µm non-fluorescent (NF) latex beads (Polysciences, Inc) were used as phagocytic targets. NF beads were co-opsonized with α-HA mAb (0.32 μg/mL) and α-FLAG mAb (2.5 μg/mL) to co-crosslink IpLITR constructs. As controls, an independent activation of the 2.6b^ITAM CYT^ and 1.1b^CYT^ constructs was performed using LY beads opsonized with α-HA mAb (0.32 μg/mL) and mouse isotype IgG1 (2.5 μg/mL) or α-FLAG mAb (2.5 μg/mL) and mouse isotype IgG1 (0.32 μg/mL). IpLITR co-expressing AD293 cells (2 × 10^5^) were seeded onto glass coverslips in a 24-well tissue culture plate and allowed to adhere overnight. Coverslips were washed with PBS and 500 µL of phagocytosis buffer containing 6 × 10^5^ opsonized NF beads was added, and the plate centrifuged at 100× *g* for 1 min at 4 °C. After 8 min at 37 °C, the cells were fixed with 4% PFA for 10 min at 37 °C and then the extracellularly exposed surfaces of the beads were stained as described [20,26]. Briefly, coverslips were placed cell-side down onto parafilm strips containing a droplet (~50 µL) of 0.5 μg/mL of Alexa 647-conjugated goat-α-mouse secondary mAb (Thermo Fisher Scientific). After 30 min at 4 °C, the coverslips were washed with antibody staining buffer (ASB; 0.05% sodium azide; 1% BSA in PBS) and then a 1× permeabilization buffer (Biolegend) was added for 15 min at room temperature. After permeabilization, the coverslips were placed onto parafilm strips containing either primary α-phosphotyrosine (1:100 *v*/*v*; Cell Signaling Technologies), α-pSHP-2 (1:50 *v*/*v*; Cell Signaling Technologies) or α-Csk rabbit pAbs (1:50 *v*/*v*; Santa Cruz Biotechnology) diluted in cell staining buffer (CSB; Biolegend). After staining for 30 min at room temperature, the cells were washed with CSB and then stained on parafilm strips with 2 μg/mL of goat-α-rabbit Alexa 488-conjugated secondary Ab (Thermo Fisher Scientific) diluted in CSB for 30 min at room temperature. Finally, the coverslips were washed in CSB and mounted onto microscope slides containing a small droplet of Prolong^®^ Gold antifade mounting media (Thermo Fisher Scientific) and allowed to cure overnight at room temperature. Imaging was performed as previously described [23,26] using a Laser Scanning Confocal Microscope (LSCM; Zeiss LSM 710, objective 60× 1.3 oil plan-Apochromat, Munich, Germany). All images were collected and analyzed using Zen 2011 software and ImageJ for calculating fluorescent intensities. Individual cellular events were then isolated from the various z-stack images and the mean fluorescent intensities (MFIs) for both bead staining and intracellular molecule (i.e., phosphotyrosine) staining were recorded. MFIs were examined for each bead–plasma membrane contact site using an ‘analysis line’, which was drawn on the z-stack images. This analysis line records MFIs for all intensities that are present along the line. To calculate integrated fluorescent intensities within phagocytic cups, a region of interest (ROI) was drawn and set consistently for all analyses. The ROI includes phagocytic cups within which the integrated fluorescent intensities were measured and quantified using ImageJ analysis software.

### 4.5. Co-Immunoprecipitation of IpLITR Constructs with Intracellular Signaling Molecules

To examine the recruitment of signaling molecules to 1.1b^WT CYT^ during receptor cross-talk, a co-immunoprecipitation assay was performed using magnetic beads as described [61]. Specifically, IpLITR co-expressing AD293 cells (i.e., 2.6b^ITAM CYT^/1.1b^WT CYT^, 2.6b^ITAM CYT^/1.1b^6YF CYT^, 2.6b^ITAM CYT^/1.1b^Prox CYT^, 2.6b^ITAM CYT^/1.1b^Distal CYT^, and 2.6b^ITAM CYT^/1.1b^3YF CYT^) were seeded (3 × 10^5^) in 24-well tissue culture plates and allowed to adhere overnight. The cell media was then aspirated and 500 μL of phagocytosis buffer containing 3 × 10^6^ magnetic beads (Thermo Fisher Scientific) co-opsonized with α-HA mAb (0.32 μg/mL) and α-FLAG mAb (2.5 μg/mL) was added to co-crosslink 2.6b^ITAM CYT^ with the various 1.1b^CYT^ constructs. Independent activation of 2.6b^ITAM CYT^ and each 1.1b^CYT^ construct was performed using magnetic beads co-opsonized with α-HA mAb (0.32 μg/mL) and isotype IgG1 (2.5 μg/mL) or α-FLAG mAb (2.5 μg/mL) and isotype IgG1 (0.32 μg/mL). The IpLITR co-expressing AD293 cells were incubated with magnetic beads opsonized with isotype IgG1 (2.82 μg/mL). Cell–bead interactions were synchronized by a 1 min 4 °C centrifugation at 200× *g*. After 8 min at 37 °C, the cells were washed with ice-cold PBS and immediately lysed in 300 μL of pre-chilled lysis buffer (50 mM Tris-HCL, 150 mM NaCl, 1% Triton X-100, protease inhibitor cocktail (Roche), phosphatase inhibitor cocktail (Sigma-Aldrich), pH = 8.0) on ice for 20 min. Magnetic beads were then isolated using a magnetic particle separator (Stemcell^TM^ Technologies) and washed twice in 500 μL of ice-cold lysis buffer before being re-suspended in 40 μL of 1X reducing buffer (Bio-Rad, Mississauga, ON, Canada) containing 5% β-mercaptoethanol (Bio-Rad). Magnetic beads were then boiled at 95 °C for 10 min to elute bound proteins. Proteins were separated via 10% SDS-PAGE gel and transferred to nitrocellulose membranes (Bio-Rad) that were probed with an HRP-conjugated α-HA mAb (1:1000) and an HRP-conjugated α-FLAG mAb (1:1000; Thermo Fisher Scientific) at 4 °C overnight to verify pull-down of the various epitope-tagged IpLITR constructs. Membranes were incubated in a stripping buffer (3.47 mM SDS, 0.2 M glycine, 1% Tween-20, pH = 2.0), blocked in 5% BSA, and re-probed with α-Csk (1:500), α-pSHP-2 (1:1000; Cell Signaling Technology), α-phosphatase and tensin homolog (PTEN 1:1000; Cell Signaling Technology) and α-Src homology 2 (SH2) domain-containing inositol 5′-phosphatase 2 (SHIP-2 1:1000; Cell Signaling Technology) mAbs at 4 °C overnight, followed by the detection of immunoreactive proteins using an HRP-conjugated goat-α-rabbit IgG (H+L) secondary mAb (1:2000; Bio-Rad) at room temperature for 2 h. Proteins were detected using SuperSignal^TM^ West Pico PLUS Chemiluminescent Substrate kits (Thermo Fisher Scientific) and imaged on a ChemiDoc imaging system (Bio-Rad).

### 4.6. Examination of Erk1/2 Pathway Activation Following the Co-Crosslinking of 2.6b^ITAM CYT^ with Various 1.1b^CYT^ Constructs

To investigate the activation of the Erk1/2 pathway following co-crosslinking of 2.6b^ITAM CYT^ and the various 1.1b^CYT^ constructs, IpLITR co-expressing AD293 cells (i.e., 2.6b^ITAM CYT^/1.1b^WT CYT^, 2.6b^ITAM CYT^/1.1b^6YF CYT^, 2.6b^ITAM CYT^/1.1b^Prox CYT^, 2.6b^ITAM CYT^/1.1b^Distal CYT^, and 2.6b^ITAM CYT^/1.1b^3YF CYT^) were seeded (3 × 10^5^) in 24-well tissue culture plates. The next day, the cell media was aspirated, and 500 μL of phagocytosis buffer containing 9 × 10^5^ YG beads co-opsonized with α-HA mAb (0.32 μg/mL) and α-FLAG mAb (2.5 μg/mL) was added. The independent activation of 2.6b^ITAM CYT^ and the various 1.1b^CYT^ constructs was also performed using YG beads opsonized with α-HA mAb (0.32 μg/mL) and isotype IgG1 (2.5 μg/mL) or α-FLAG mAb (2.5 μg/mL) and isotype IgG1 (0.32 μg/mL). Cell–bead interaction was synchronized by a 1 min 4 °C centrifugation at 100× *g* and then the cells were incubated at 37 °C for 2 min, 8 min, 15 min and 30 min. After each incubation, the cells were immediately lysed in 150 μL of pre-chilled lysis buffer on ice for 20 min and whole cell lysates were collected and subjected to two rounds of sonication (50 kHz, 3 s/round). After sonication, the lysates were centrifuged at 13,200× *g* for 10 min at 4 °C and 12.5 µL of cleared lysate was mixed with 50 µL of 4× reducing buffer containing 5% β-mercaptoethanol. SDS-PAGE and Western blot were performed as described earlier. The membranes were first probed with an α-pErk1/2 pAb (1:1000; Cell Signaling Technology, Danvers, MA, USA) overnight at 4 °C, followed by incubation with an HRP-conjugated goat-α-rabbit IgG (H+L) secondary mAb (1:2000) at room temperature for 2 h. The membranes were incubated in a stripping buffer (3.47 mM SDS, 0.2 M glycine, 1% Tween-20, pH = 2.0), re-blocked with 5% BSA, and re-probed with an α-Erk1/2 mAb (1:1000; Cell Signaling Technology) overnight at 4 °C, followed by incubation with an HRP-conjugated goat-α-mouse IgG (H+L) secondary pAb (1:2000) at room temperature for 2 h.

### 4.7. Statistics

Data is represented throughout as mean ± SEM. Experimental groups were compared using a one-way ANOVA, followed by the Tukey test using Prism 6 software (GraphPad Software, La Jolla, CA, USA). Letters (e.g., a, b, and c) indicate statistical significance (*p* ≤ 0.05) between means as indicated in each figure.

## Supplementary Materials

Supplementary materials can be found at https://www.mdpi.com/1422-0067/21/14/5146/s1.

## Abbreviations

ASB	antibody staining buffer
BSA	bovine serum albumin
Csk	C-terminal src kinase
CYT	cytoplasmic tail
Erk1/2	extracellular signal-regulated kinase 1/2
FcRs	Fc receptors
FcRLs	FcR-like proteins
FBS	fetal bovine serum
HA	hemagglutinin
HRP	horseradish peroxidase
IpLITRs	*Ictalurus punctatus* leukocyte immune-type receptors
ITAM	immunoreceptor tyrosine-based activation motif
ITIM	immunoreceptor tyrosine-based inhibitory motif
ITSM	immunoreceptor tyrosine-based switch motif
LILRs	leukocyte Ig-like receptors
LRC	leukocyte receptor complex
LY	light-yellow
MFIs	mean fluorescent intensities
NF	non-fluorescent
PFA	paraformaldehyde
YF	tyrosine to phenylalanine
PTEN	phosphatase and tensin homolog
PLCγ1	phospholipase Cγ1
ROI	region of interest
SHIP-2	Src homology 2 domain containing inositol 5′-phosphatase
SHP-2	Src homology 2-containing protein tyrosine phosphatase 2
TLRs	toll-like receptors
TM	transmembrane
YG	yellow-green
