# Supplementary material for: A Fish Leukocyte Immune-Type Receptor Uses a Novel Intracytoplasmic Tail Networking Mechanism to Cross-Inhibit the Phagocytic Response
Source: Int J Mol Sci. 2020 Jul 21;21(14):5146. doi: 10.3390/ijms21145146 (PMC7404264; doi:10.3390/ijms21145146)
Supplement: Supplementary file 1 [file ijms-21-05146-s001.pdf]

Suppl. Figure 1.

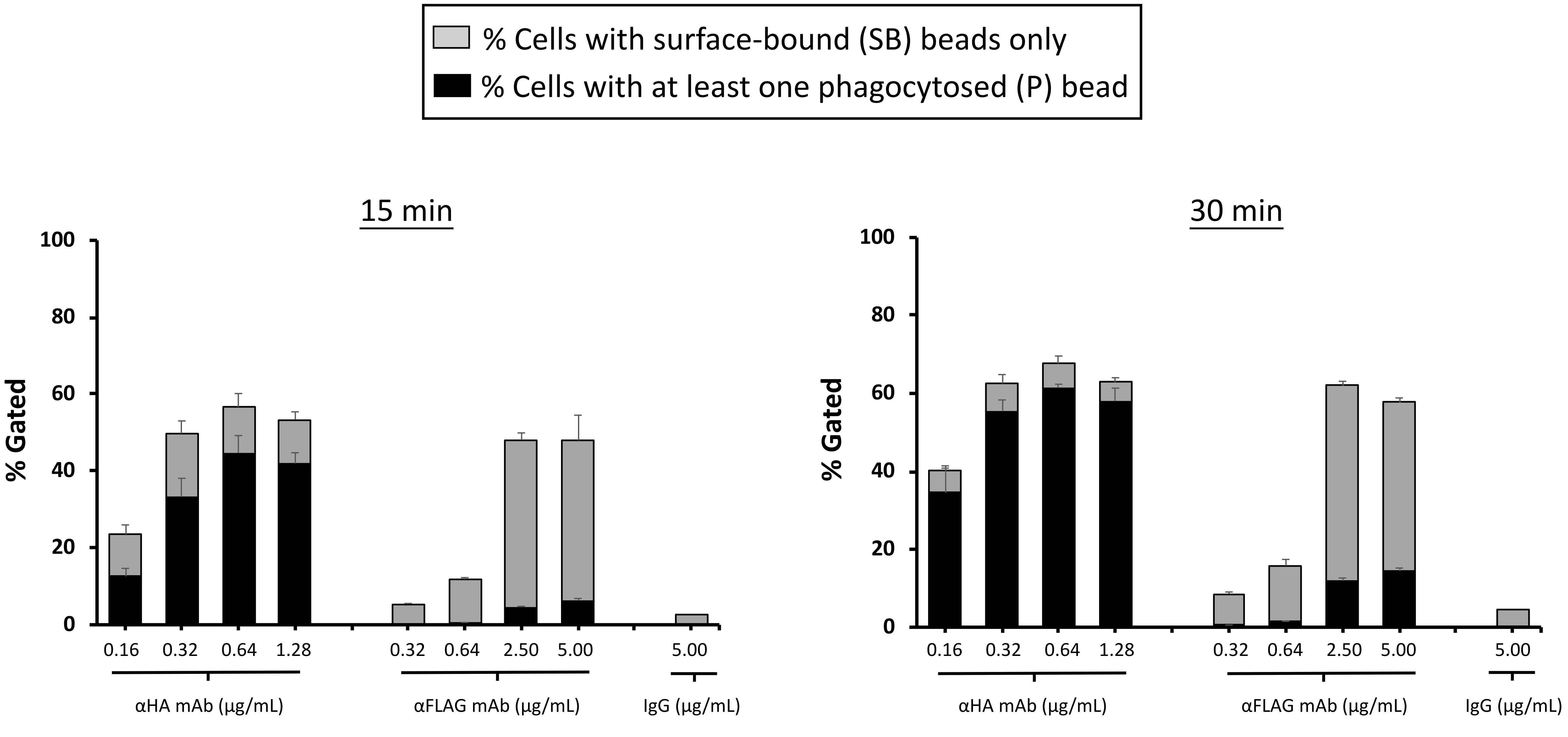

Suppl. Figure 2.

A.

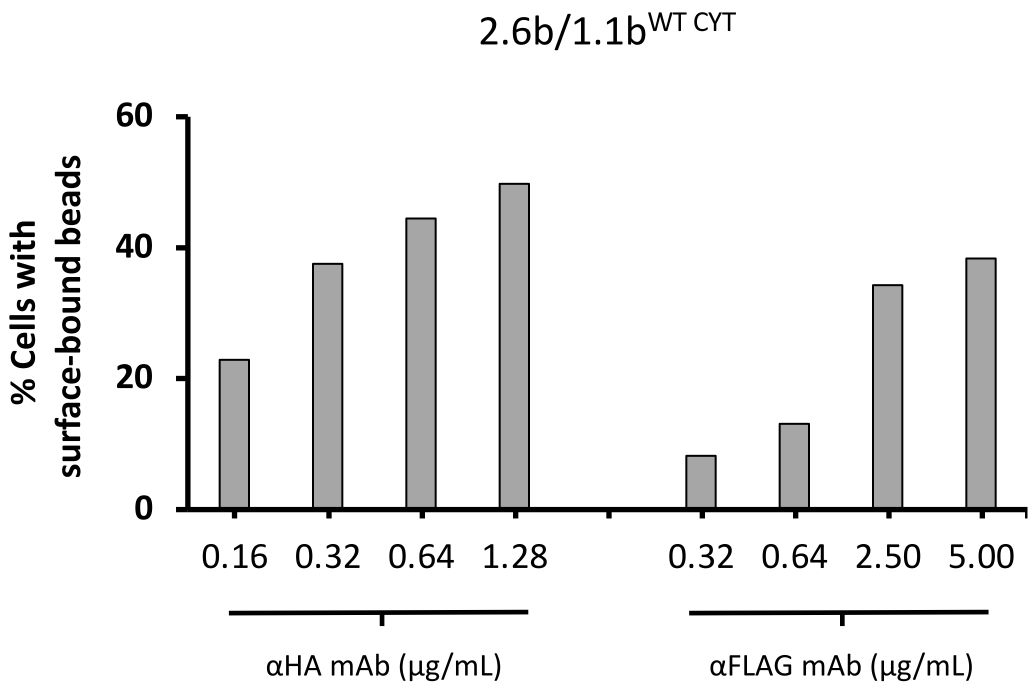

B.

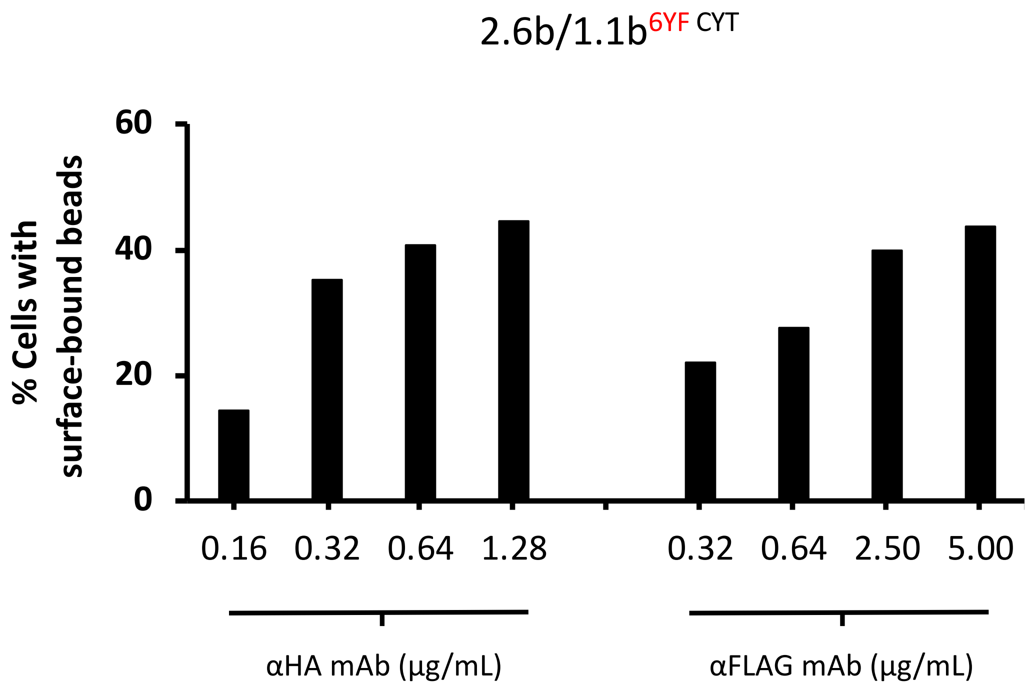

C.

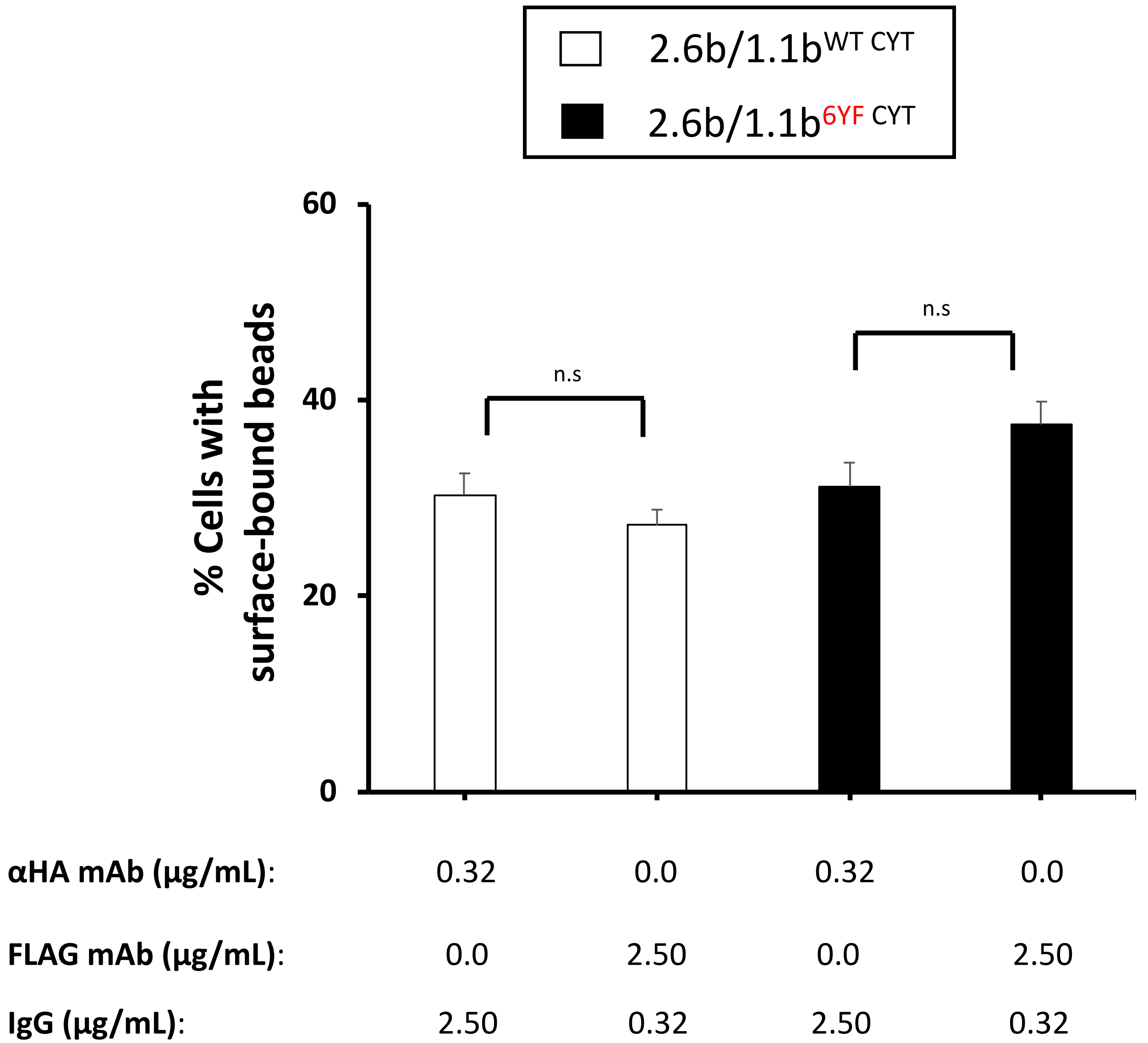

2.6b/1.1b WT CYT
2.6b/1.1b 6YF CYT
% Cells with surface-bound beads
60
40
20
0
n.s
n.s
αHA mAb (µg/mL): 0.32 0.0 0.32 0.0
FLAG mAb (µg/mL): 0.0 2.50 0.0 2.50
IgG (µg/mL): 2.50 0.32 2.50 0.32

A.

B.

Suppl. Figure 3.

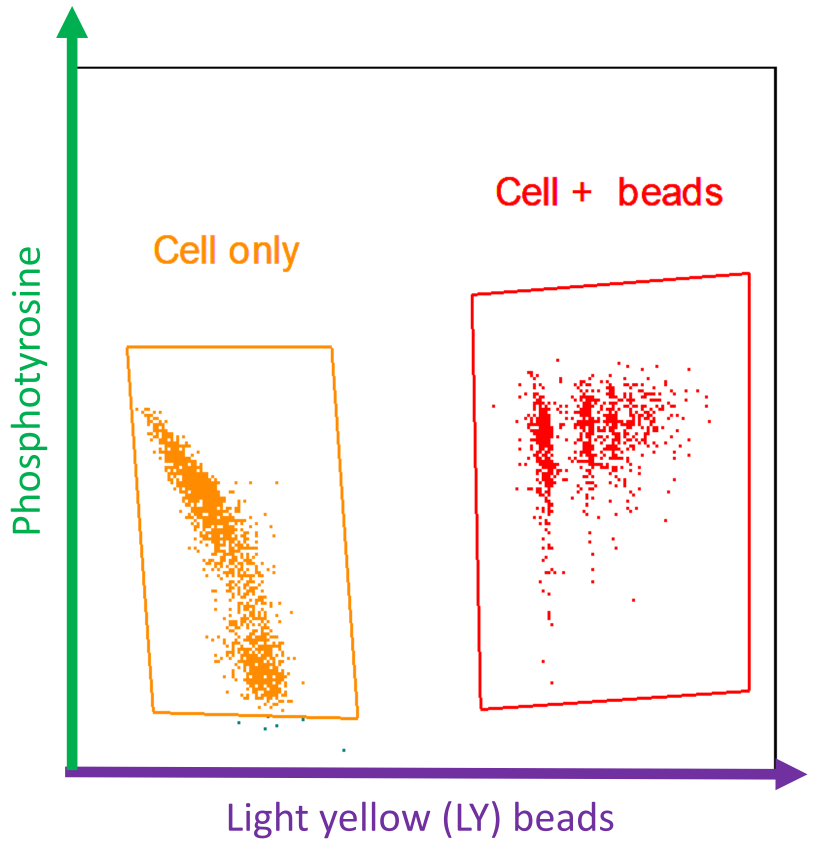

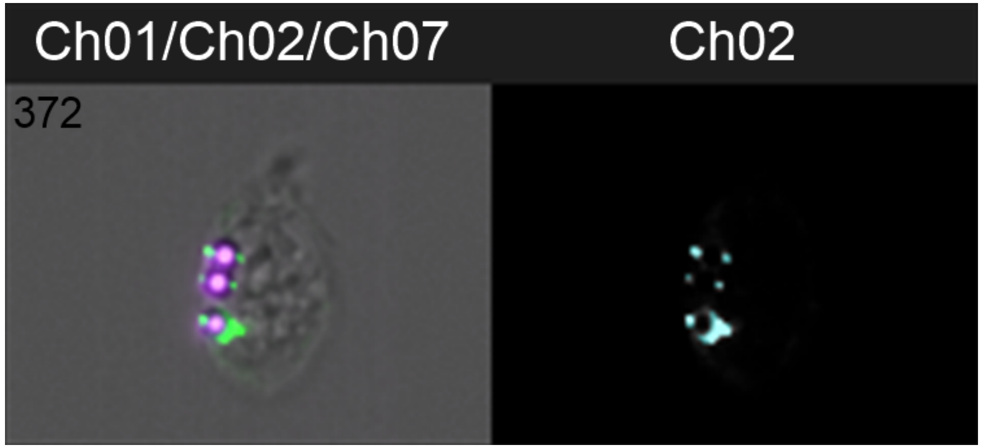

**Mask**	**Description**
Intensity (M02, 72-4025)	Intensity mask used to identify phospho-tyrosine signals above background in channel 2

C.

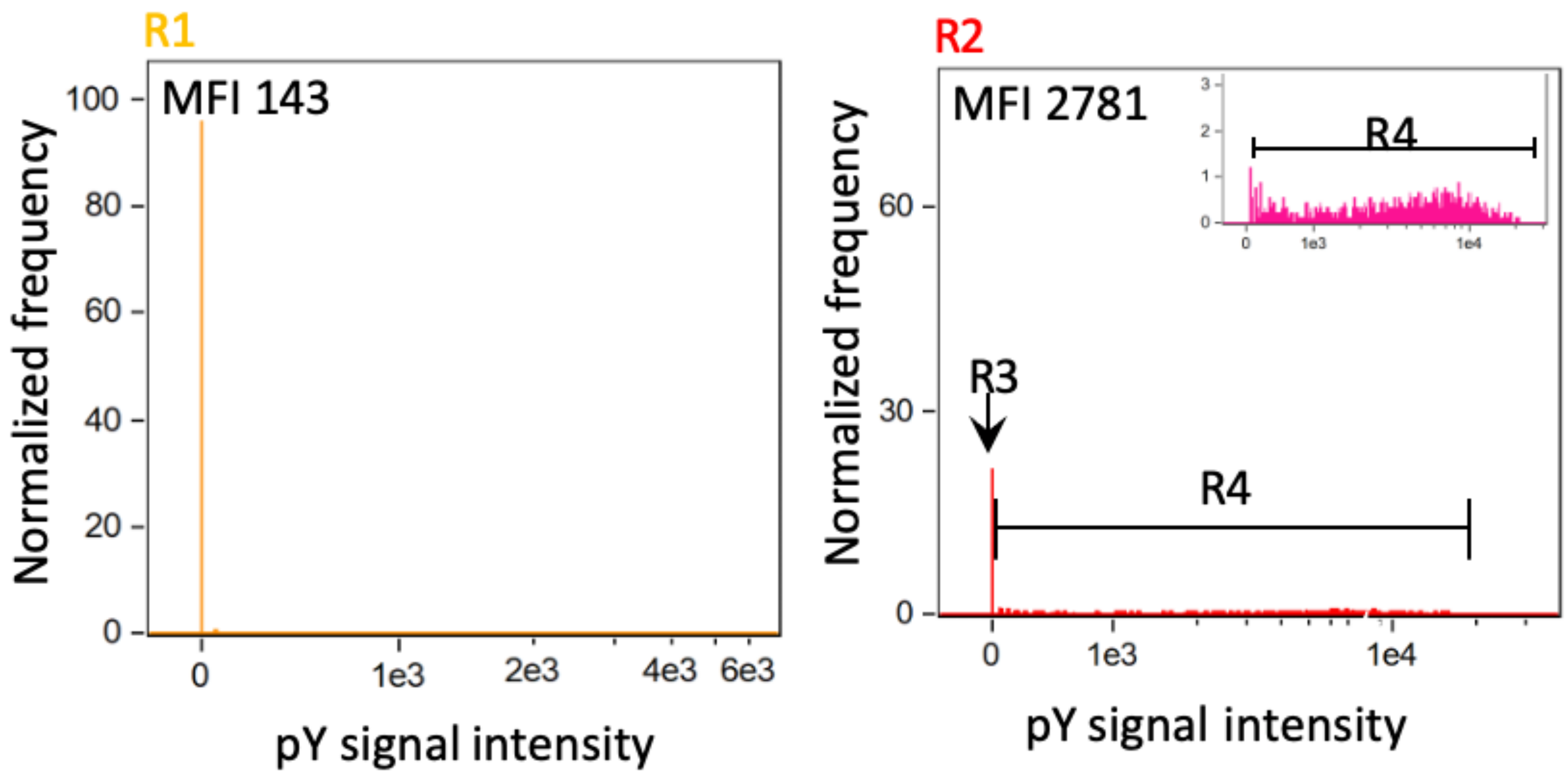
R1
MFI 143
Normalized frequency
100
80
60
40
20
0
0
1e3
2e3
4e3
6e3
pY signal intensity
R2
MFI 2781
Normalized frequency
60
30
0
R3
R4
0
1e3
1e4
pY signal intensity
R4
3
2
1
0
0
1e3
1e4
